# Testing Late Bronze Age mobility in southern Sweden in the light of a new multi-proxy strontium isotope baseline of Scania

**DOI:** 10.1371/journal.pone.0250279

**Published:** 2021-04-21

**Authors:** Pernille Ladegaard-Pedersen, Serena Sabatini, Robert Frei, Kristian Kristiansen, Karin Margarita Frei

**Affiliations:** 1 National Museum of Denmark, Copenhagen, Denmark; 2 Department of Historical Studies, University of Gothenburg, Gothenburg, Sweden; 3 Department of Geosciences and Natural Resource Management, University of Copenhagen, Copenhagen, Denmark; 4 Globe Institute, Lundbeck Foundation, GeoGenetics Centre, Copenhagen, Denmark; Museo delle Civiltà, ITALY

## Abstract

The Bronze Age of Sweden’s southernmost region, Scania, is complex and intriguing. One could say that Scania represented in many ways a gateway for people, ideas and material culture connecting continental Europe with Sweden. Shedding light on the dynamics of human mobility in this region requires an in depth understanding of the local archaeological contexts across time. In this study, we present new archaeological human data from the Late Bronze Age Simris II site, located in an area of Scania showing a dynamic environment throughout the Late Bronze Age, thus likely involving various forms of mobility. Because the characterization of solid strontium isotope baselines is vital for delineating human mobility in prehistory using the strontium isotope methodology, we introduce the first environmentally based multi-proxy (surface water-, plant- and soil leachates) strontium isotope baselines for sub-regions of Scania. Our results show, that the highly complex and spatially scattered lithologies characterising Scania does not allow for a spatially meaningful, geology-based grouping of multi-proxy data that could be beneficial for provenance studies. Instead, we propose sub-regional baselines for areas that don’t necessarily fully correspond and reflect the immediate distribution of bedrock lithologies. Rather than working with a Scania-wide multi-proxy baseline, which we define as ^87^Sr/^86^Sr = 0.7133 ± 0.0059 (n = 102, 2σ), we propose sub-regional, multi-proxy baselines as follows: Area 1, farthest to the north, by ^87^Sr/^86^Sr = 0.7184 ± 0.0061 (n = 16, 2σ); Area 2, comprising the mid and western part of Scania, with ^87^Sr/^86^Sr = 0.7140 ± 0.0043 (n = 48, 2σ); Area 3–4, roughly corresponding to a NW-SE trending zone dominated by horst-graben tectonics across Scania, plus the carbonate dominated south western part of Scania with ^87^Sr/^86^Sr = 0.7110 ± 0.0030 (n = 39, 2σ). Our results also reflect that the complexity of the geology of Scania requires systematic, high density, statistically sound sampling of multiple proxies to adequately constrain the baseline ranges, particularly of those areas dominated by Precambrian lithologies. The averaging effect of biosphere Sr in surface water might be beneficial for the characterization of baselines in such terranes. Our sub-regional, area-specific baselines allow for a first comparison of different baseline construction strategies (single-proxy versus multi-proxy; Scania-wide versus sub-regional). From the Late Bronze Age Simris II site, we identified six individuals that could be analysed for Sr isotopes, to allow for an interpretation of their provenance using the newly established, environmental strontium isotope baselines. All but one signature agrees with the local baselines, including the ^87^Sr/^86^Sr value we measured for a young individual buried in a house urn, typically interpreted as evidence for long distance contacts. The results are somewhat unexpected and provides new aspects into the complexity of Scandinavian Bronze Age societies.

## Introduction

Scania encompasses the south-easternmost part of the Scandinavian Peninsula. Its strategic geographical position dominating the access to the Baltic Sea has played a crucial role in the history of the region from the earliest colonization at the end of the last glacial period until modern times. Archaeological remains that expand from the Late Palaeolithic, through the Stone, Bronze and Iron Ages until the Middle Ages allow reconstructing of a dynamic picture of the local prehistory and early history. The known evidence consists of a rich variety of sites, including prehistoric settlements as well as complex urban milieus, military fortresses, monuments, and ritual and funerary contexts from all periods [1–12]. Such multifarious archaeological evidence shows strong links with traditions and innovations from the rest of the continent, suggesting that the inhabitants of the region were actively participating in exchange networks with other neighbouring Scandinavian groups as well as with distant communities from the continent. Several studies have been undertaken to investigate mobility of both prehistoric and medieval humans and animals from this region, some using the strontium isotope tracing system [13–17]. Those studies provide a wide overview on the mobility of individuals and animals from the Late Palaeolithic, the Neolithic, the Early Bronze Age, the Iron Age and the Middle Ages.

Modern mobility studies of prehistoric individuals using strontium isotopes are dependent on the establishment of relevant bioavailable strontium isotope baselines. Strontium is a trace element, with four naturally occurring isotopes (^84^Sr, ^86^Sr, ^87^Sr, ^88^Sr) out of which three are stable and one (^87^Sr) is radiogenic, derived from the decay of ^87^Rb. The ^87^Sr/^86^Sr ratio varies between bedrocks, dependent on age and the Rb/Sr concentration of the rocks, of which the latter is largely determined by rock type [18,19]. Strontium is released from bedrock, soil and atmospheric deposits through weathering processes. The dissolved and mobilised strontium fractions enter the food chain of humans and animals through drinking water and plant-based food [20,21]). The bioavailable fraction of strontium entering the food chain is strongly dependent on geographical area. Bioavailable, i.e. mobile, strontium is taken up by plants from the soil pore water through their root systems [18,20–22] and while the ^87^Sr/^86^Sr ratios remain constant along the pathway from bedrock to the food-chain, it couples a strontium isotope signature of plant-based food to a specific geographic location and soil property [20,21,23]. Strontium in surface waters (rivers, creeks, lakes, ponds) and spring waters, which constitute drinking water sources used by humans and animals, represents an average value of strontium from the catchments of rivers and aquifers. The strontium isotope signatures of these waters are ultimately dependent on the soil and bedrock geology of the respective catchments, and on the weathering dependent reaction pathways within these, and consequently will tie their isotope signatures to the geological background of a specific geographic area.

This work presents the results of strontium isotope analyses carried out on six individuals and additionally analyses of two faunal remains from the Late Bronze Age cemetery of Simris II in south-eastern Scania. When investigating mobility within the gateway region of Scania, the site of Simris II is of special interest based on archaeological finds of burial urns with a particular shape that suggest acquaintance with long-distance networks. Furthermore, results from Simris II provide the first set of data from the key period at the beginning of the first millennium BCE, which has not been investigated to date. To this aim we constructed the first comprehensive, environmentally-based, Scania-wide, multi-proxy (surface water, plants and soil leachates) strontium isotope baseline. The results presented herein enables us to critically discuss the mobility expressed by the Simris II samples.

## Previous strontium isotope results from Scania and neighbouring regions

**Fig 1:**
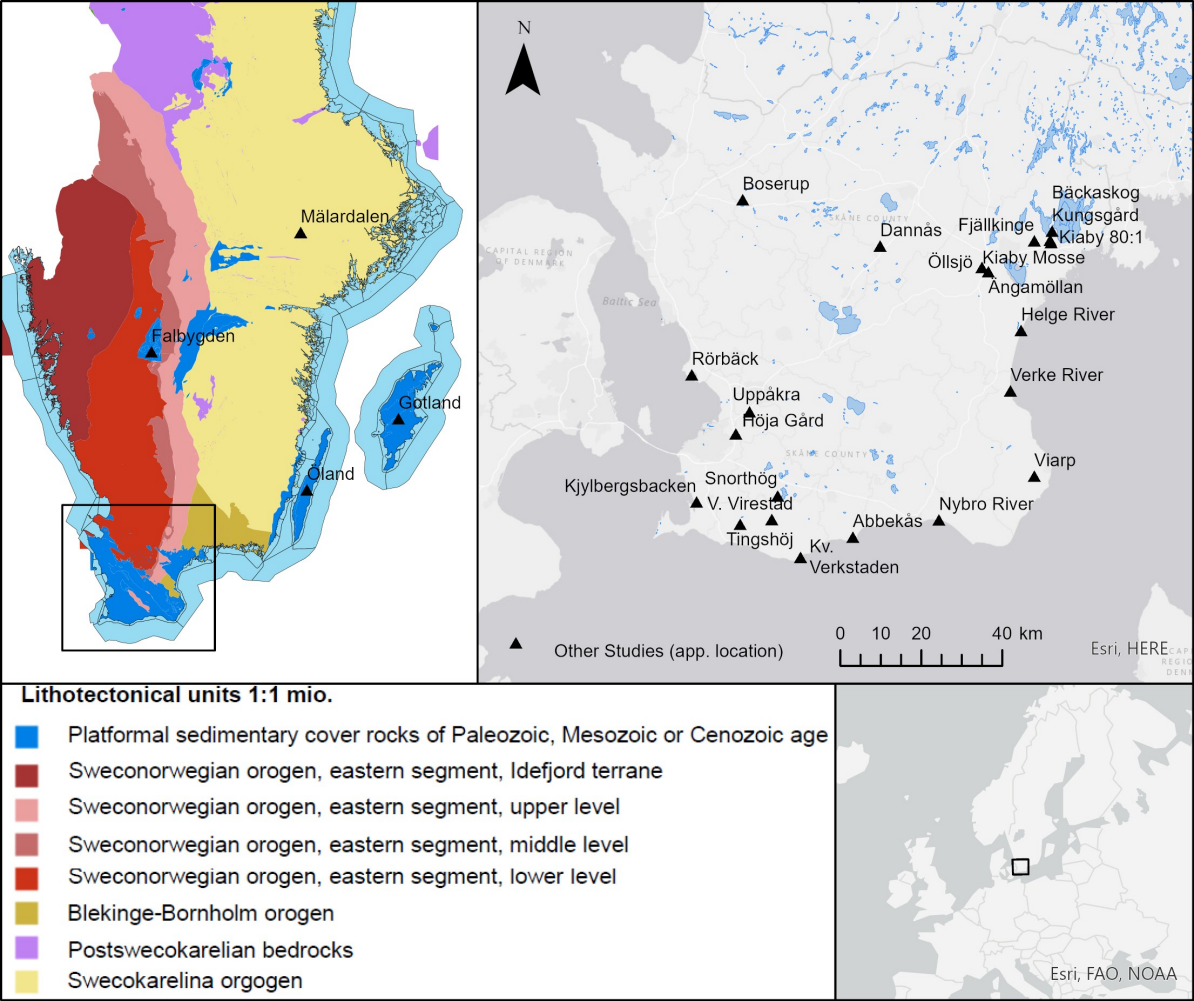
Scania, Southern Sweden. Upper left corner: Lithotectonical units of Scania and southern Sweden, based on the thematic, publically available 1:1 mio map from the Geological Survey of Sweden (www.Sgu.se). Approximate locations of sites from other studies mentioned in text [13,15,24–28]. Lower right corner showing study area on a European scale for orientation purposes. Maps generated using ESRI ArcGIS Pro software and basemaps, licensed to the National Museum of Denmark.

So far, few studies report bioavailable strontium isotope ratios in environmental proxies (water, soil or plants) from a few areas within Scania and neighbouring areas. These include reports of ^87^Sr/^86^Sr ratios in natural stream waters discharging from Swedish and Finnish mainland and into the Baltic Sea (summarized in [24,25]). Rivers running through areas dominated by Paleozoic, Mesozoic and Cenozoic sediments (Fig 1), as prevalent in the south of Sweden, have high concentrations of Sr (100–500 μg L^-1^) and are characterised by low ^87^Sr/^86^Sr ratios, close to that of the modern seawater ratio of ^87^Sr/^86^Sr = 0.7092. Alternatively, rivers that stay in the Precambrian shield areas (Fig 1) have lower Sr concentrations (15–50 μg L^-1^) and exhibit higher ^87^Sr/^86^Sr ratios between 0.721–0.745 [24,25]. Relevant to the baseline of Scania are the following three streams, which we extracted from [24,25]: 1) The Nybro River, which runs through areas dominated by Mesozoic limestone and clay, shale, sandstone and coal, and discharges at the southern coast with a ^87^Sr/^86^Sr ratio of 0.7111 (Sr concentration 234 μg L^-1^); 2) The Verke River, which runs through both Precambrian granodioritic-granitic gneiss terranes and coastal Cambrian sandstones and shales near Kivik on the eastern coast, with a ^87^Sr/^86^Sr ratio of 0.7130 (Sr concentration 77 μg L^-1^); and 3) the Helge River, which runs through Mesozoic lime- and sandstone in the Kristianstad area and discharges on the eastern coast near Yngsjö, with a ^87^Sr/^86^Sr ratio of 0.7161 (Sr concentration 58 μg L^-1^) (Fig 1).

Strontium isotope signatures of fauna for baseline reference purposes were also reported. In a mobility study of the rich Iron Age settlement of Uppåkra situated in south-west Scania near the modern city of Lund, Price [15] reports four strontium isotope signatures of faunal remains from the following sites: Kv. Verkstaden (^87^Sr/^86^Sr = 0.7105), Fjällkinge (^87^Sr/^86^Sr = 0.716), Dannås (^87^Sr/^86^Sr = 0.716) and Boserup (^87^Sr/^86^Sr = 0.711) (Fig 1). These were used as reference baseline values in the study by Price [15], reporting the strontium isotope composition of tooth enamel of ten humans, along with tooth enamel of 14 locally excavated cattle and six locally excavated pigs from within the site of Uppåkra, which were also used as reference baseline values [15]. By comparing the range of ^87^Sr/^86^Sr = 0.7111 to 0.7191 (mean = 0.7132; ± 0.0024, 1σ) for the humans in their study with that of ^87^Sr/^86^Sr = 0.7097 to 0.7134 (mean = 0.7118; ±0.0009, 1σ) for tooth enamel of the cattle, and ^87^Sr/^86^Sr = 0.7113 to 0.7118 (mean = 0.7115; ±0.0002, 1σ) for tooth enamel of the pigs, Price [15] could identify several non-local individuals from Uppåkra in Scania. Also Arcini [29] reported a compilation of faunal enamel (both domesticated animals such as cows and pigs, and wild animals such as rodents, a fox and a roe deer) from all over Sweden. Out of this dataset 46 analyses are from samples from within the Scania region, including the fauna data from [15]. The faunal analyses from Scania define a mean of 0.712 ± 0.004 (2σ, n = 46). In a recent study, ^87^Sr/^86^Sr ratios of individuals from Late Neolithic to Early Bronze Age are reported [13]. This study includes data from 16 individuals from the Late Neolithic I (2350–1950 BC), 7 individuals from the Late Neolithic II (1950–1700 BC), two individuals from the transitional period (Late Neolithic to Early Bronze Age, and 36 Early Bronze Age (1700–1100 BC) individuals from southern Sweden [13]. The individuals were excavated from the sites of Bäckaskog Kungsgård, Kiaby, Kiaby Mosse, Öllsjö, Ângamöllan, Viarp, Rörbäck, Höja Gård, Kjyllbergsbacken, Snorthög, Tingshøj, V. Virestad and Abbekås (Fig 1). At the time of the study, due to the lack of a well-defined and large-coverage bioavailable strontium baseline for the region, these authors used a tentative bioavailable ^87^Sr/^86^Sr baseline range between ~0.708 and 0.713 [13], which they based on the geology of the area, and previously reported faunal values [14–17,30].

Other archaeological studies focused on the region north and north-east of Scania. In a study focusing on Iron Age migration on the Swedish island of Öland, located off the east coast of southern Sweden in the Baltic sea (Fig 1), Wilhelmson and Ahlström [31] compiled strontium isotope ratios of faunal samples (enamel from archaeological mammals and/or shells of modern snails) available at that time from southern Sweden. For the island of Öland itself, which has a predominantly Paleozoic limestone basement, these authors reported a strontium isotope baseline of ^87^Sr/^86^Sr = 0.7140 ± 0.0024 (1σ; n = 25). In this very study focusing on Öland, these authors also report a fauna-based baseline for the island of Gotland (Fig 1), likewise dominated by Paleozoic sediments (limestone, marl and sandstone), which they define as ^87^Sr/^86^Sr = 0.7106 ± 0.0003 (1σ; n = 7). Fraser and co-workers [32] added ten soil samples and five fauna samples from the island of Gotland to the Wilhelmson and Ahlström [31] data set, which resulted in an expansion of the baseline for Gotland to ^87^Sr/^86^Sr = 0.7120 ± 0.0018 (1σ; n = 26).

In contrast to these limestone and soft-sediment dominated areas, Wilhelmson and Ahlström [31] reported a baseline for the mainland site of Mälardalen in the region of Stockholm (Fig 1), an area dominated by crystalline basement rocks. A strontium isotope baseline of ^87^Sr/^86^Sr = 0.7335 ± 0.0070 (1σ; n = 4) reported by these authors, differs from baselines with relatively low ^87^Sr/^86^Sr values in the above mentioned islands as well as other parts of mainland south Sweden.

Finally, a recent study focused on another area in southern Sweden with a bedrock geology partially comparable to parts of the Scania region. This is the area of Falbygden in central southwestern Sweden (Fig 1), which has been focus of several mobility studies using strontium isotopes [33–35]), including a study producing a comprehensive baseline based on ^87^Sr/^86^Sr ratios of 67 samples (61 stream water samples and five samples from archaeological fauna) [28]. This latter study was combined with previous measurements of two water samples and 21 tooth enamel samples of archaeological, non-domestic animals [28]. This study is important, as it finds many parallels, especially in terms of tectonically juxtaposed, geologically very different terranes and lithologies. Like in Scania, the bedrock geology in the area of Falbygden, characterised by smaller and restricted outcrop areas is dominated by Paleozoic sediments within otherwise Precambrian crystalline rocks pertaining to the Eastern segment of the Sveconorwegion orogeny. Blank and co-workers [28] found areas with Paleozoic sedimentary rocks to produce significantly lower bioavailable ^87^Sr/^86^Sr ratios ranging from 0.7119–0.7191, with a mean of 0.7146 ±0.0014 (1σ; n = 45), compared to areas with Precambrian crystalline rocks, which have ^87^Sr/^86^Sr ratios ranging from 0.7154–0.7280, with a mean of 0.7220 ±0.0032 (1σ; n = 39) [28].

Bulk rock strontium isotope compositions of crystalline Precambrian rocks in Southern Sweden, compiled by [35]), reflect the expanded, comparatively large ^87^Sr/^86^Sr range of bioavailable signatures to be expected from areas dominated by the Precambrian granite-gneiss terranes in Sweden. Sjögren and co-workers [35] report bulk rock ^87^Sr/^86^Sr values from the Sveconorwegian Eastern segment to range from ^87^Sr/^86^Sr = 0.708 to 1.206 (mean 0.801 ± 0.143; 1*σ*; n = 56), reflecting a high isotopic variability and a strontium isotope value distribution strongly skewed towards higher, more radiogenic ^87^Sr/^86^Sr values [35].

The above mentioned studies and existing data sets set the framework for our attempt to establish a bioavailable strontium isotope baseline for entire Scania for which a comprehensive and spatially representative baseline is still lacking. Consequently, our goal is to construct the first multi-environmental proxy-based strontium isotope baseline for this region. For this purpose, we collected and analysed water, soil leachates and plant samples from sites carefully chosen to reflect the lithological variation of country rocks present. This approach was recently successfully applied in e.g. France [36], in Cyprus [37] and on the Peloponnese, Greece [38]. Our sampling sites, which covers the major lithologies of Scania, were chosen to allow for 1) the baselines to be used as relevant reference for their application in provenance studies of prehistoric individuals excavated from archaeological sites within Scania; 2) the exploration of potential sub-regional variations of bioavailable strontium isotope ranges in Scania; 3) the distinction of regional inter-relationships between the different environmental proxy archives.

## Archaeology of Simris II

**Fig 2:**
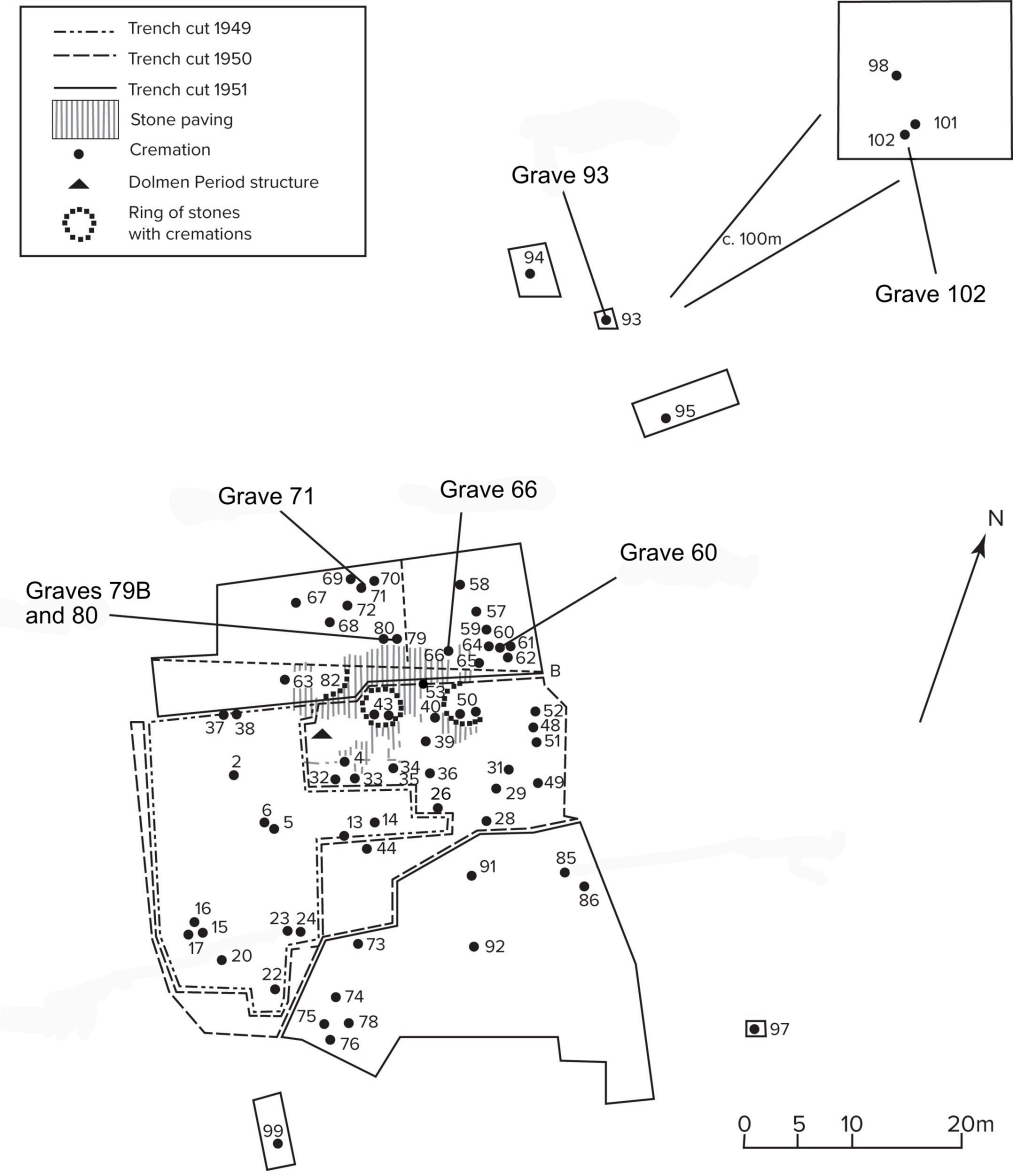
Excavation plan of the Late Bronze Age cemetery Simris II. The graves, from which the individuals sampled in the present study were excavated, are marked.

The cemetery at Simris II was accurately excavated between 1949 and 1951 [39,40] (Fig 2). The site lies a few km from today’s coastline, on a relatively high ground west of the modern town of Simris. It was in use over a long period of time and features burials dated from the Late Bronze Age until the Roman Iron Age.

The Late Bronze Age cemetery was primarily in use during the Nordic Period V (c. 900–700 BCE), with some contexts dating to the end of Period IV (c. 1100–900 BCE) and possibly some to Period VI (c. 700–500 BCE, cf Stjernquist 1961, 122). A total of 78 individuals were unearthed at Simirs II [39,41]; they were buried in 65 different contexts, including 61 simpler burials in urns, cremation graves also known as cremations pits, three burial monuments with multiple burials inside (stone circles 43, 50 and 82) and a collective grave with three individuals, each in its own urn (grave 49). Two of the Period V graves (23 and 71) suggest acquaintance with long-distance networks and exchange practices due to the shape of the burial urns [39,42]. A so-called door urn, and a face/door urn were found in grave 71 and 23, respectively. Both items are considered expressions of the northern European house urn phenomenon [42]. House urns are burial urns shaped as buildings/houses or as vases with architectural features such as roofs and doors. The phenomenon emerges at the end of the second millennium BCE to last for a few generations. The distribution pattern of these house urns includes northern Poland, northern and former eastern Germany, Denmark, south and eastern Sweden. It has been argued that the house urns in northern Europe were inspired by and expresses a link to the roughly contemporary so-called hut urn tradition from the central western regions of the Italian Peninsula [42,43]. On the other hand, the face features on the face/door urn from grave 23 provide links to the so-called face urns, which was another phenomenon that was widespread across northern Europe and Poland [44] and not originally local to Scania.

As in most parts of the continent at the end of the second millennium BCE, the earlier Bronze Age custom to bury the deceased in inhumation graves is abandoned in favour of the crematory ritual [45]. Several authors have suggested that with the passage from inhumation to cremation, Bronze Age burial urns acquired an important role materially embodying the deceased now transformed in ashes eventually displaying features which were able to evoke the identity or the social status of the deceased or of the keen/family group to which they belonged [46,47]. The presence of exceptionally shaped urns, such as those in graves 23 and 71, strengthens the idea that the community burying its deceased at Simris II was acquainted with, or directly linked to, long distance networks of various sizes and characteristics and that it was considered relevant to mark such links in the burial arena [39,48].

## Geology and glaciation of Scania

**Fig 3:**
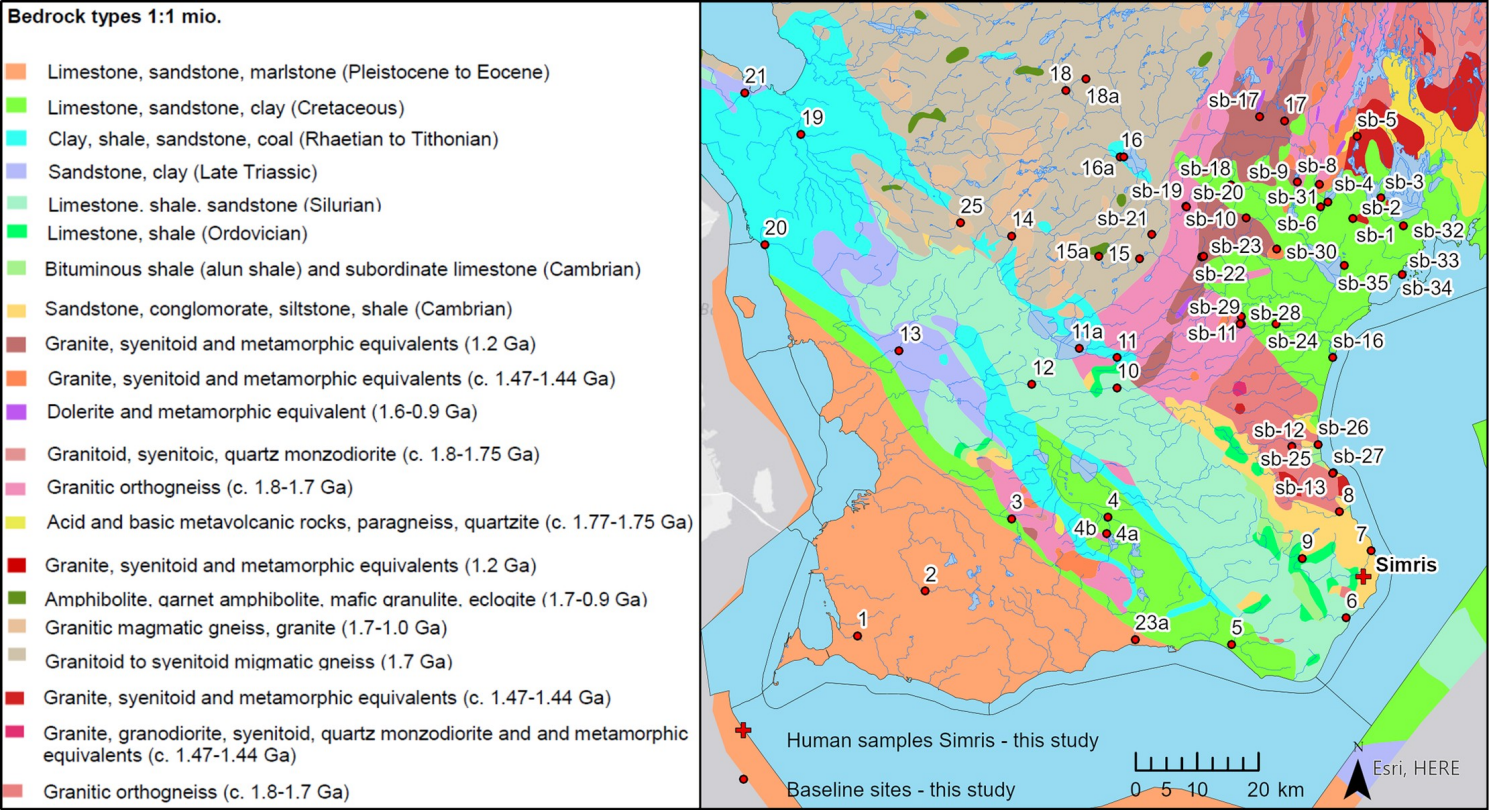
Baseline sampling sites of the present study, and the approximate location of the Late Bronze Age cemetery Simris II. Maps showing bedrock types and river networks of Scania extracted from the thematic 1:1 mio maps publically available from the Geological Survey of Sweden (www.sgu.se), and the Swedish Meteorological and Hydrological Institute (www.smhi.se). Maps generated using ESRI ArcGIS Pro software, licensed to the National Museum of Denmark.

Scania forms the southernmost part of Sweden (Fig 1). The geology of the province is shaped by tectonic displacements and faulting that has led to the exposure of differently aged bedrock and sediments in the region (e.g. [13,49–51]). The bedrock geology of the north-eastern part of Scania is dominated by Precambrian granites and gneisses of the Fennoscandian Shield, belonging mostly to the eastern segment of the Sveconorwegian orogen, with few areas belonging to the Blekinge-Bornholm orogeny (Fig 1). The south-western part of Scania is dominated by platform and slope sediments deposited during the Paleozoic, Mesozoic and Cenozoic (Fig 1). In the north-east of Scania, west of the town of Kristianstad, Mesozoic limestone and sandstone sequences overlay the crystalline Precambrian basement rocks (Fig 3). In the Tornqvist zone, an approximately 50 km wide seismically active transect running NW-SE, the landscape is dominated by horst and graben structures, with several NW–SE running ridges formed by Precambrian rocks (Fig 3). Within the transect, Paleozoic sediments such as shales, limestones and sandstones, are exposed alongside younger Mesozoic sediments, such as lime-, sand-, mud- and claystones. Part of the older sedimentary sequences are intruded by Carboniferous dolerite dykes. The area south-west of the tectonic transect is dominated by Mesozoic and Cenozoic lime-, sand- and marlstone, and closely resembles the Pre-Quaternary successions in neighbouring country Denmark (Fig 3).

**Fig 4:**
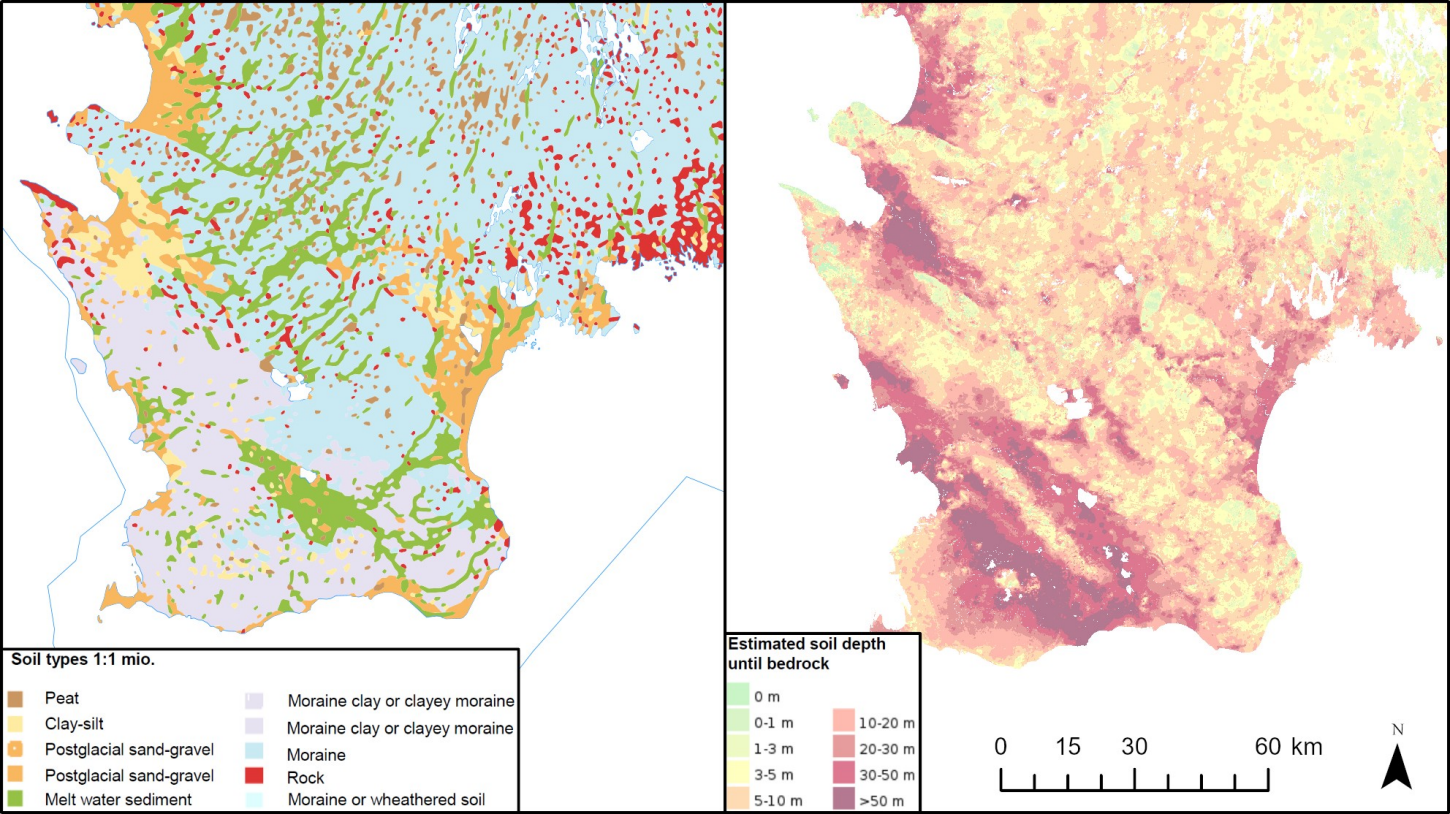
Maps showing soil types and estimated soil depth of Scania. Maps are extracted from the thematic 1:1 mio maps publically available from the Geological Survey of Sweden (www.sgu.se), and generated using ESRI ArcGIS Pro software, licensed to the National Museum of Denmark.

Scania was glaciated repeatedly during the Pleistocene, leaving the landscape covered by glacial deposits of varying thickness and composition [15,49] (Fig 4). In Scania, tills deposited from ice lobes running from north to north-east, and thus carrying material dominantly from areas covered by crystalline Precambrian rocks, can be separated from tills deposited by ice moving in from the south, and thus carrying material dominated by the carbonate rich sedimentary rocks of the Baltic [52]. Most glaciogenic sediments of Scania are moraines, dominated by clayey moraines to the south and west, and sandy moraines to the north and east. Glacial meltwater deposits and postglacial sand and gravel outwash deposits also occupy larger areas. Areas with exposed bedrock not overlain by glaciogenic sediments are predominantly found to the north and east (Fig 4). Soil depth from terrain surface to the subsurface bedrock is varying, with soil thicknesses of more than 50 m in the south-west and central parts, to 3–5 m in coastal zones and in parts to the north and northeast (Fig 4). The south-western part of Scania is dominated by agriculture, whereas the north-eastern part is predominantly forested.

## Materials and methods

### Sampling and samples

From the Bronze Age site of Simris II, remains from six individuals were chosen for analysis. All but one human sample are cremated petrous bone (otic capsule), representing early childhood stages from prenatal to approximately two years of age [53]. The last human sample is cremated dental enamel from either M1 or M2. Petrous bone has been shown not to remodel after the age of c. 2 years, and to act as a substitute sampling material for provenance investigations based on strontium isotope analysis of burned human remains [53]. Additional experimental studies aiming at investigating potential contamination in bone material showed that “in vivo strontium isotopic ratios are retained in calcined bone” [54]. Recently, this methodology was applied in cremated human bone remains from the Neolithic site of Stonehenge in England to investigate potential mobility [55]. Yet another recent study, from individuals excavated from a Bronze Age cemetery in Germany points to that cremated dental enamel also retain its strontium isotope ratios despite heat-alteration and long-term deposition in the soil [56]. Additional to the human samples, two faunal, cremated petrous bones, possibly from lamb, were analysed. Permissions from the Lund University Historical Museum were granted to conduct destructive analysis on the archaeological animal samples.

Additionally, a total of 106 samples (29 stream water samples, 8 lake water samples, 24 soil samples and 45 plant leaf samples) were collected from Scania in June 2017 (sampling sites 1–23), as well as in May and August 2017 (sampling sites sb-1-sb-35). The sampling sites were selected to cover the distinct lithological formations of Scania (Fig 3). To the greatest extent, pristine sites were sampled, and agricultural soils were avoided. Where sampling had to be done near farmed areas, samples were taken from uncultivated border zones. From 22 sampling sites (labelled 1–21, 23), stream water, soil and plant samples were collected from an area within a radius of maximum 200 m.

Water was sampled in 50 mL sampling tubes. Topsoil samples were taken from 10–15 cm below soil surface, or 10–15 cm below the mor layer, where such was present. Leaves were sampled from bushes, as previously shown suitable for baseline investigation by [57], and collected for Sr baselines in comparative multi-proxy studies in Cyprus [37] and the Peloponnese, Greece [38]. Bushes with a total height of 2–4 m were preferred and the leaf sampling height was preferably chosen to be approximately 1.5 m above ground. Leaves were cleaned using a moist, clean towel to remove dust particles, and left to air-dry overnight.

Sampling sites assigned a single number denote the sites where multi-proxy sampling (stream water, soil and plant) was performed. Sampling sites labelled with ‘a’ is a lake sample. The ‘sb’-samples denote the sites where plant samples were primarily collected, but in some cases were complemented by an additional water or soil sample. These sites are shown as eg. sb- 8+9, and the proxy type of the respective additional sample from such a site is given in Table 1. No specific permissions were required for the collection of water, soil and plant samples, according to Swedish legislation.

**Table 1:** Strontium isotope ratios of human and faunal remains of the Simris II site.

Sample specifics	Sample type	^87^Sr/^86^Sr	Error, 2SE	Osteological information
Grave 60	Petrous bone L	0.71182	0.00001	Adult, Male
Grave 66	Petrous bone R	0.71177	0.00001	Adult, Female
Grave 80	Petrous bone L	0.71191	0.00002	Adult, Female
Grave 71	Cremated tooth enamel (M1 or M2)	0.71177	0.00001	c. 15 years
Grave 93	Petrous bone L*	0.71505	0.00001	Adult, Male (2 adult male indiv. in the urn)
Grave 102	Petrous bone L	0.71223	0.00001	c. 10–15 years
Faunal, grave 79 B	Petrous bone 3	0.71179	0.00001	-
Faunal, grave 79 B	Petrous bone 2	0.71225	0.00001	-

*Possible traces of middle ear infections suggested by S. Scott Reiter during sampling at the National Museum of Denmark, but not further verified.

### Laboratory procedures

Sample procedures were performed inside an over-pressured clean room at either the National Museum of Denmark, Brede, or at the Department of Geosciences and Natural Resource Management at the University of Copenhagen.

#### Sample preparation

The surfaces of the petrous bone material were carefully cleaned mechanically to remove the dirt. Using pre-cleaned dental drills, samples were collected from the petrous bones (otic capsule), discarding the initial drill dust to allow for the sampling of 1 mg of non-contaminated drill dust. The pre-cleaned samples were dissolved in conc. HCl and conc. H_2_O_2_, and evaporated down for Sr separation. The enamel of one tooth was carefully cleaned mechanically to remove dirt, dentine and dental calculus, and 1–2 mg of enamel was sampled, dissolved in conc. HCl and conc. H_2_O_2_, and evaporated down for Sr separation.

Water aliquots of 5 mL from each water sample were transferred to 7 mL pre-cleaned Teflon beakers (Savillex^TM^) and left to evaporate on a hot plate at 100°C for 3–4 hours, after which an additional 5 mL from the same samples were transferred into the Teflon beakers and left to evaporate (between 4 hours or overnight), in order to allow for a total water sample volume of 10 mL.

When producing soil leachates, air dried soil samples were thoroughly mixed and approximately 5 g of each sample (avoiding roots and organic material) were gently ground in an agate mortar. One gram of homogenized soil was weighed and transferred to a 15 mL Sarstedt centrifuge tube, and 5 mL of a 1M NH_4_NO_3_ solution was then added to the tube. Tubes were fastened in a LLG uniROTATOR 2, rotated at 70 rpm for 2 hours, left to settle for 1 hour and centrifuged for 10 minutes at 3000 rpm. 2.5 mL of the supernatant were pipetted off and transferred to 7 mL Teflon beakers (Savillex^TM^) and evaporated on a hot plate at 100°C. Selected samples were spiked with a ^84^Sr enriched tracer solution prior to their transfer into Teflon beakers. This allowed us to determine the Sr concentration ([Sr]) by isotope dilution. The leaching agent was chosen to be unbuffered NH_4_NO_3_ (DIN V 19730, German Institute for Standardisation), following the lead of [58], thus ensuring comparability between strontium isotope baseline studies.

From sampling sites 1–21 and 23, four to five leaves from the air-dried plant sample, were ground in an agate mortar to tea-leaf consistency or finer. 100 mg of each sample was weighed into pre-cleaned Alsint 99.7 (Haldenwanger) crucibles and incinerated at 750°C for 3 hours. Ashes were transferred to 7 mL Teflon (Savillex^TM^) beakers using 1 mL of ultrapure water from an Elga Purelab Flex System (18.2 MΩ cm), then evaporated, re-dissolved in 1 mL concentrated HNO_3_ (Sea Star), and evaporated again. From sampling sites sb-1, sb-4-sb-7, sb-9-sb-13, sb-16-sb-19, sb-21-sb-22, sb-23-sb-25, sb-29-sb-30, sb-32, and sb-34-35, air dried plant samples were crushed to tea-leaf or smaller consistency in an agate mortar, and 100 mg plant material was dissolved in Teflon vials using alternating additions of conc. HNO_3_ (Sea Star) and conc. H_2_O_2_ (Sea Star), and increasing temperatures (from initial cold digestion to allow for initial reaction up to 70°C) until the appearance of clear or pale yellow liquids, that were evaporated for Sr separation.

Evaporated samples were taken up in a few drops of 3 M HNO_3_ and loaded on disposable columns made from Eppendorf^TM^ 1mL pipette tips. These were mounted with a pre-cleaned, pressed-in filter and pre-cleaned in double-distilled 6 M HCl. Prior to sample loading, the columns were charged with 7 drops (app. 200 μL) of mesh 100–150, intensively pre-cleaned SrSpec (Eichrome Inc./Triskem) resin. The Sr elution recipe was modified from Horowitz et al. [59], following the lead of [60]. Strontium was stripped using ultrapure water (Elga Purelab Flex System (18.2 MΩ cm)) and the final solutions were dried down on a hotplate at 100°C.

#### Analytical procedures

Samples were dissolved in 2.5 μL of a Ta_2_O_5_-H_3_PO_4_-HF activator solution and loaded directly on previously outgassed 99.98% single Re filaments. Samples were measured at 1250–1300°C in dynamic multi-collection mode on a Thermal Ionisation Mass Spectrometer (TIMS; VG Sector 54 IT mass spectrometer equipped with eight Faraday detectors; Department of Geoscience and Natural Resource Management, University of Copenhagen). Five nanogram loads of the NBS 987 Sr standard gave ^87^Sr/^86^Sr = 0.710238 ± 0.000020 (n = 5, 2σ). The ^87^Sr/^86^Sr values of the samples were corrected for the offset relative to the NBS 987 value of 0.710245 [61]. The errors reported in Table 1 are within-run precisions (two standard errors; 2se) of the individual runs. Procedure blanks were run with the samples and yielded <30 pg of Sr and ^87^Sr/^86^Sr ratios of ~0.7085 -~0.7096. The amount of blank Sr is insignificant relative to the amount of sample Sr (typically tens of nanograms or higher), and therefore, the measured Sr isotopic compositions of the multi-proxy samples were insensitive in the critical first five digits to a blank correction.

## Results and discussion

### Human samples from Simris II

The sample collection was constrained by factors such as availability and preservation of bone material. It was therefore not possible to analyse all the contexts that we originally intended to sample, including the female individual in grave 23 buried in the face/door urn. By chance, the graves that were analysed (60, 66, 71, 79, 80, 93 and 102) happen to be topographically quite close to each other, located in the northern part of the cemetery, as we know it today (Fig 2). Four samples come from a dense cluster of graves bordering the stone paved area, which supposedly formed the central part of the cemetery [39]. The remaining two contexts (graves 93 and 102) come from two different areas c. 50 m (grave 93) and c. 150 m to the north-east of the cemetery (grave 102), which suggests that the original size of the burial ground might have been considerably larger than the excavation plan (Fig 2). From a demographic point of view, the samples include both male and female adult individuals and young individuals (Table 1). Two faunal petrous bones, possibly from lamb, from grave 79 were also analysed.

### Environmental strontium isotope ranges

**Fig 5:**
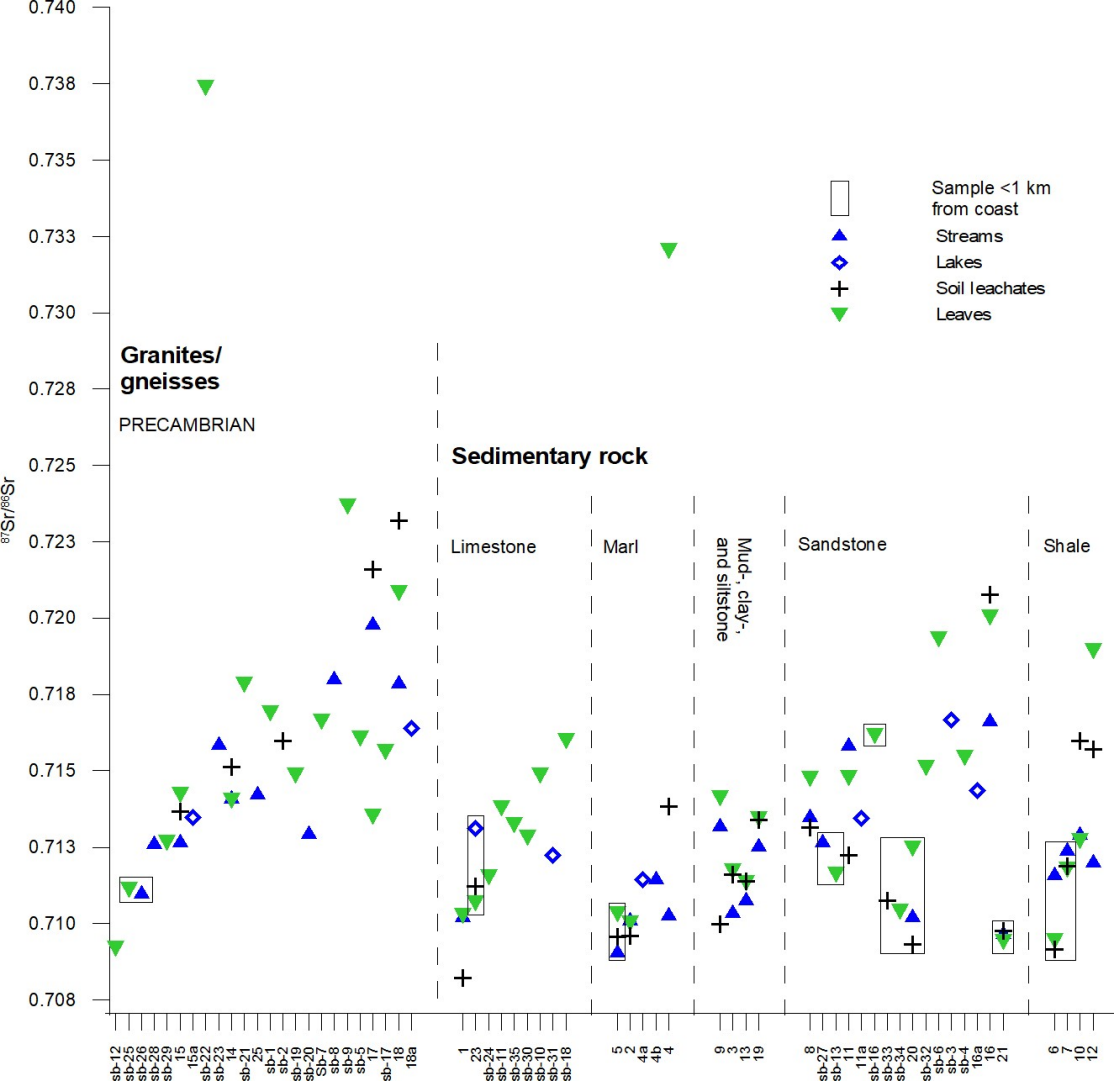
Lithology-based grouping of strontium isotope ratios (^87^Sr/^86^Sr) of samples. Sites are grouped according to the lithology of the underlying bedrock at the sampling site. Groups are not spatially coherent. Within each group, sampling sites are sorted with increasing latitude from left to right. Sampling sites located within 1 km of the nearest coast are indicated by a rectangle. Analytical errors, as specified in Table 2, are within individual markers.

Results of the bioavailable ^87^Sr/^86^Sr ratios in surface water (stream water and lakes), soil leachates and plants are reported in Table 2 alongside site information, and presented in a graph as single measurements (Fig 5). Geological classification of data is adapted from the high resolution geological maps (1:50:000–1:250.000) publically available from the Geological Survey of Sweden. These data reveal a range of strontium isotopic ratios from a minimum of ^87^Sr/^86^Sr = 0.7082 (soil leachates, site 1) to a maximum of ^87^Sr/^86^Sr = 0.7374 (plants, site sb-22). The dataset contains two samples, which have strontium isotopic ratios markedly higher than the remaining samples. These are the plant samples from site sb-22: ^87^Sr/^86^Sr = 0.7374 and site 4: ^87^Sr/^86^Sr = 0.7320 (Fig 5). The plant sample from site 4 was collected from an area dominated by Cretaceous marls (Table 2). The soil leachate and stream water analysed from the same site yielded significantly less radiogenic ^87^Sr/^86^Sr ratios of 0.7138 and 0.7103, respectively (Fig 5). The nearby Sövdesjön (site 4a) revealed a water ^87^Sr/^86^Sr ratio of 0.7114, and Klingvalsåen (site 4b), a creek draining from the lake towards site 4, also, as expected, yielded a ^87^Sr/^86^Sr ratio = 0.7114 (Fig 5). It is unclear at this stage how to explain the vastly more radiogenic value measured in the particular plant sample with the very high ^87^Sr/^86^Sr signature, but the bush from which the leaves were sampled was growing in the vicinity to an asphalt road supported by a stabilizing granite/gneiss gravel foundation. We can speculate that a release of strontium from this crystalline gravel bed not local to the site could contaminate the local biosphere Sr signatures with radiogenic Sr in the root system of the particular plant sampled there. We therefore chose to remove the sample from the dataset prior to statistical analysis. The plant sample from site sb-22, located in the Precambrian granite terrain near Djurröd (Table 2), is sampled near the water sample sb-23 (Djurröd stream), showing a much lower strontium isotopic ratio of ^87^Sr/^86^Sr = 0.7158 (Fig 5). Outliers like are not surprising in areas dominated by Precambrian crystalline bedrock. As mentioned in the previous section, a wide range of bulk rock Sr isotope signatures from ^87^Sr/^86^Sr = 0.708 to 1.206 (mean 0.801 ± 0.143; 1*σ*; n = 56) have been reported for gneisses and granites from the Sveconorwegian Eastern segment [35], which is the dominant Precambrian terrane in Scania. It illustrates the high variability of Precambrian bulk rock compositions and their skewed distributions towards higher, i.e., more radiogenic ^87^Sr/^86^Sr values [35]. Given these large variations in bulk rock Sr isotope signatures from this terrane, the Sr signature of sample sb-22 could be explained by local outcrop geology, in this case dominated by an elevated Sr isotope signature of the country rocks in this particular location.

**Table 2:** Bioavailable strontium isotope ratios of waters, soil leachates and plants from Scania.

Site no	Location	Water body	Northing	Easting	Geology	Period	Streams		Lakes		Soil leachates		Plants	
					(SGU 1:50.000–1:250.000)	(SGU 1:50.000–250.000)	^87^Sr/^86^Sr	±2SE	^87^Sr/^86^Sr	±2SE	^87^Sr/^86^Sr	±2SE	^87^Sr/^86^Sr	±2SE
sb-12	Brösarps Backar		55° 43’ 13.01"	14° 7’ 38.25"	Granodioritic-granitic gneiss	Precambrian							0.70918	0.00001
sb-25	Rävlunda		55° 43’ 26.89"	14° 11’ 41.08"	Granodioritic-granitic gneiss	Precambrian							0.71111	0.00001
sb-26	Rävlunda	Haväng stream	55° 43’ 26.89"	14° 11’ 41.08"	Granodioritic-granitic gneiss	Precambrian	0.71096	0.00001						
sb-28	Maltesholm	Maltesholm spring	55° 53’ 57.33"	13° 59’ 26.53"	Gneiss	Precambrian	0.71258	0.00001						
sb-29	Maltesholm		55° 53’ 57.33"	13° 59’ 26.53"	Gneiss	Precambrian							0.71265	0.00001
15	Häglinge	Vrams stream	55° 59’ 29.04’’	13° 40’ 25.32’’	granitic gneiss	Precambrian	0.71265	0.00002			0.71367	0.00001	0.71423	0.00001
15a	Tjörnarp Sjö		55° 59’ 29.04’’	13° 40’ 25.32’’	granitic gneiss/grabboid-dioritoid	Precambrian			0.71348	0.00002				
sb-22	Djurröd	* *	55° 59’ 45.97"	13° 53’ 8.09"	Granite	Precambrian							0.73735	0.00001
sb-23	Djurröd	Djurröds stream	55° 59’ 45.97"	13° 53’ 8.09"	Granite	Precambrian	0.71584	0.00001						
14	Djupdalsmölle	Rönne stream	56° 01’ 16.32’’	13° 23’ 15.6’’	Granitic gneiss	Precambrian	0.71408	0.00001			0.71511	0.00001	0.71402	0.00013
sb-21	Ljungarum		56° 01’ 40.2"	13° 45’ 16.11"	Granitic gneiss	Precambrian							0.71782	0.00001
25	Söderåsen	Skär stream	56° 02’ 18.47’’	13°15’12.56’’	Granite	Precambrian	0.71423	0.00001						
sb-1*	Fjälkinge backa	* *	56° 03’ 19.65"	14° 16’ 40.2"	Granite	Precambrian							0.71688	0.00002
sb-2*	Fjälkinge backa	* *	56° 03’ 19.65"	14° 16’ 40.2"	Granite	Precambrian					0.71598	0.00001		
sb-19	Nävlinge		56° 04’ 09.49"	13° 50’ 32.53"	Granodioritic-granitic gneiss	Precambrian							0.71485	0.00002
sb-20	Nävlinge	Nävlinge stream	56° 04’ 09.49"	13° 50’ 32.53"	Granodioritic-granitic gneiss	Precambrian	0.71293	0.00000						
sb-7	Balsberget		56° 06’ 17.2"	14° 11’ 22.98"	Granite	Precambrian							0.71662	0.00001
sb-8	Torsebro	Helge stream	56° 06’ 26.14"	14° 7’ 56.9"	Monzodiorite-granodiorite	Precambrian	0.71800	0.00001						
sb-9	Torsebro		56° 06’ 26.14"	14° 7’ 56.9"	Monzodiorite-granodiorite	Precambrian							0.72366	0.00001
sb-5	Arkelstorp		56° 10’ 30.45"	14° 17’ 15.74"	Granodiorite granite	Precambrian							0.71605	0.00001
17	Knislinge	Helge stream	56° 11’ 46.74’’	14° 5’ 47.82’’	Granite	Precambrian	0.71976	0.00002			0.72159	0.00001	0.71350	0.00001
sb-17	Frännarp	* *	56° 12’ 07.84"	14° 1’ 52.63"	Granite	Precambrian							0.71561	0.00001
18	Röke	Röke stream	56° 14’ 08.34’’	13° 31’ 18.18’’	Granodioritic-granitic gneiss	Precambrian	0.71786	0.00001			0.72320	0.00001	0.72081	0.00001
18a	Humlesjön	Humlesjön (Lake)	56° 14’ 08.34’’	13° 31’ 18.18’’	Granodioritic-granitic gneiss	Precambrian			0.71639	0.00001				
6	Skillinge		55° 28’ 15.72’’	14° 16’ 21.12’’	Shale	Silurian	0.71156	0.00001			0.70914	0.00001	0.70942	0.00000
9	Listarum	Komstad stream	55° 33’ 25.02’’	14° 9’ 24.12’’	Mudstone, siltstone, claystone	Ordovician	0.71317	0.00002			0.70997	0.00001	0.71412	0.00001
7	Simrishamn	Tommarp stream	55° 34’ 11.64’’	14° 20’ 5.16’’	Shale	Cambrian	0.71238	0.00002			0.71187	0.00001	0.71177	0.00001
8	Rörum	Rörums Södra stream	55° 37’ 35.1’’	14° 15’ 5.76’’	Sandstone	Cambrian	0.71349	0.00002			0.71314	0.00001	0.71473	0.00001
sb-13**	Kiviksröset		55° 40’ 58.33"	14° 14’ 7.02"	Sandstone	Cambrian							0.71159	0.00001
sb-27**	Kiviksröset	Spring	55° 40’ 58.33"	14° 14’ 7.02"	Sandstone	Cambrian	0.71265	0.00001						
10	Korsholm	Brå stream	55° 48’ 08.4’’	13° 40’ 11.94’’	Shale	Silurian	0.71289	0.00001			0.71597	0.00002	0.71270	0.00001
12	Hurva	Brå stream	55° 48’ 17.1’’	13° 26’ 57.6’’	Shale	Silurian	0.71197	0.00002			0.71570	0.00001	0.71892	0.00001
5	Nybrostrand	Karbusa stream	55° 25’ 47.0’’	013° 58’ 40.7’’	Marl	Creataceous	0.70905	0.00001			0.70957	0.00001	0.71030	0.00001
3	Gennarp	Höje stream	55° 36’ 27.7’’	013° 24’ 16.8’’	Mudstone, claystone, siltstone	Creataceous	0.71033	0.00001			0.71159	0.00001	0.71170	0.00001
4	Ilstorp	Drainage channel	55° 36’ 46.7’’	013° 39’ 13.2’’	Marl	Creataceous	0.71026	0.00001			0.71383	0.00001	0.73201	0.00001
4b	Sövde	Klingvalsåen	55° 35’20.85’’	013° 39’ 2.26’’	Marl	Creataceous	0.71142	0.00002						
4a	Sövde	Sövdesjön (Lake)	55° 36’ 46.7’’	013° 39’ 13.2’’	Marl	Creataceous			0.71143	0.00002				
11	Hörby	Hörby stream	55° 50’ 47.04’’	13° 40’ 9.18’’	Sandstone	Jurassic	0.71580	0.00002			0.71223	0.00001	0.71475	0.00001
11a	Öster Ringsjön	Öster Ringsjön (Lake)	55° 50’ 47.04’’	13° 40’ 9.18’’	Sandstone	Jurassic			0.71346	0.00002				
13	Norrvindinge	Sax stream	55° 50’ 57.06’’	13° 6’ 6.18	Mudstone, claystone, siltstone	Triassic	0.71075	0.00002			0.71137	0.00001	0.71133	0.00001
sb-16	Frieseboda		55° 51’ 5.26"	14° 13’ 50.96"	Sandstone	Cretaceous							0.71614	0.00001
sb-24	Everöd		55° 53’ 59.09"	14° 4’ 53.2"	Limestone	Cretaceous							0.71152	0.00001
sb-11	Västra Vram	* *	55° 54’ 35.53"	13° 59’ 25.54"	Limestone	Cretaceous							0.71379	0.00001
sb-33	Landö		55° 58’ 26.3"	14° 24’ 30.73"	Sandstone	Cretaceous					0.71076	0.00001		
sb-34	Landö		55° 58’ 26.3"	14° 24’ 30.73"	Sandstone	Cretaceous							0.71040	0.00001
20	Helsingborg	Råå stream	55° 59’ 53.88’’	12° 44’ 27.9’’	Sandstone	Jurassic	0.71020	0.00001			0.70933	0.00001	0.71246	0.00001
sb-35	Rinkaby		55° 59’ 9.26"	14° 15’ 25.02"	Limestone	Cretaceous							0.71324	0.00001
sb-30	Öllsjö		56° 0’ 34.15"	14° 4’ 49.1"	Limestone	Cretaceous							0.71281	0.00001
sb-32	Gualöv		56° 02’ 43.35"	14° 24’ 41.79"	Sandstone	Cretaceous							0.71509	0.00001
sb-10	Önnestad		56° 03’ 15.54"	13° 59’ 58.32"	Limestone	Cretaceous							0.71486	0.00002
sb-6	Lundahögerna	* *	56° 04’ 16.85"	14° 11’ 37.36"	Sandstone	Cretaceous							0.71930	0.00001
sb-31	Råbelövsjön	Råbelövsjön (Lake)	56° 04’ 44.01"	14° 12’ 47.06"	Limestone	Cretaceous			0.71223	0.00000				
sb-3	Ivösjön	Ivösjön (Lake)	56° 05’ 7.57"	14° 21’ 5.97"	Sandstone	Cretaceous			0.71666	0.00001				
sb-4	Ivösjön		56° 05’ 7.57"	14° 21’ 5.97"	Sandstone	Cretaceous							0.71543	0.00002
sb-18	Vinslöv		56° 06’ 5.41"	13° 57’ 32.13"	Limestone	Cretaceous							0.71598	0.00001
16***	Mjölkalunga	Mjölkalungan stream	56° 08’ 24.06’’	13° 40’ 2.46’’	Sandstone	Jurassic	0.71661	0.00002			0.72078	0.00001	0.72002	0.00001
16a***	Finjasjön	Finjasjön (Lake)	56° 08’ 24.06’’	13° 40’ 2.46’’	Sandstone	Jurassic			0.71435	0.00001				
19	Strövelstorp	Vege stream	56° 09’ 40.50’’	12° 49’ 46.02’’	Mudstone, claystone, siltstone	Jurassic	0.71250	0.00001			0.71339	0.00000	0.71342	0.00000
21	Jonstorp	Görlev stream	56° 13’ 9.54’’	12° 40’ 43.8’’	Arkose (sandstone)	Triassic	0.70964	0.00001			0.70974	0.00001	0.70939	0.00001
1	Räng		55° 25’ 49.4’’	013° 00’ 52.6’’	Limestone	Tertiary	0.71019				0.70820	0.00001	0.71025	0.00001
23	Svartadammen	Skönedals pond	55° 26’ 05.9’’	013° 43’ 47.8’’	Limestone	Tertiary			0.71312	0.00001	0.71122	0.00001	0.71066	0.00001
2	Ll. Svedala	Sege stream	55° 29’ 55.0’’	013° 11’ 12.4’’	Marl	Tertiary	0.71009	0.00001			0.70958	0.00001	0.71001	0.00001

* = sample site located in ~1 km^2 granite outcrop from the Blekinge-Bornholm orogen, surrounded by sand- and limestone. of Cretaceous age.

** = sample site located near the border to mudstone, claystone, siltstone of Cambrian age.

*** = sample site located in ~21 km^2 outcrop of Jurassic sandstone, surrounded by granodioritic-granitic gneiss from the Sveconorwegian orogeny.

Sites grouped according to geological era. Within each era, sites are sorted according to degree north. The classification of the bedrock geology is done from high resolution geological maps (1:50.000–250.000) publically available from the Geological Survey of Sweden (www.sgu.se).

**Fig 6:**
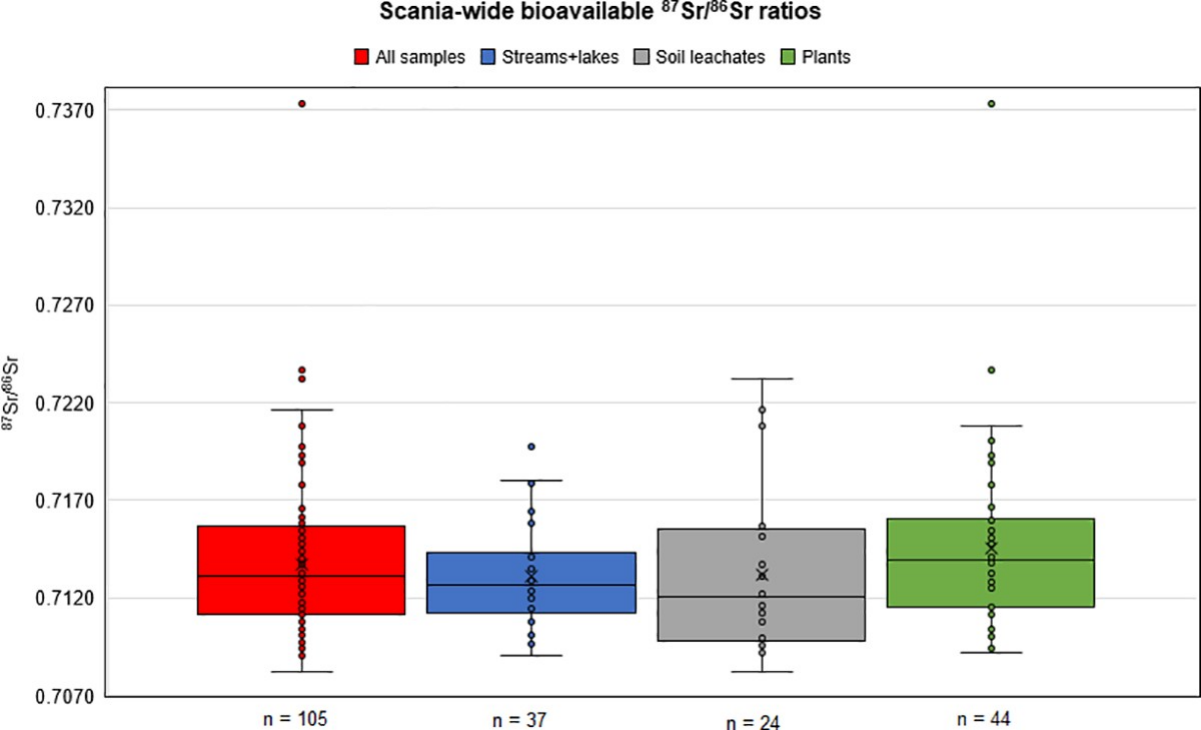
Scania-wide bioavailable strontium isotope data presented in box-and-whisker plots. Data are contained in Table 3. The plant sample from site 4 is not included in the respective plot due to suspected contamination, see discussion in text. X in box = mean. Middle line in box = median. Bottom line of box = 1^st^ quartile (Q1). Top line of box = 3^rd^ quartile (Q3). Whiskers extend from minimum to maximum values. Outliers = A data point exceeding a distance of 1.5 times the interquartile range (IQR = Q3-Q1) below or above the 1^st^ quartile or 3^rd^ quartile range.

The distribution of bioavailable ^87^Sr/^86^Sr ratios from Scania are presented as box-and-whisker diagrams for each proxy in Fig 6 (excluding the plant sample from site 4). The box-and-whisker plots show that the sample populations of each proxy type, as well as the entire proxy data set, have a tendency to be skewed towards higher ^87^Sr/^86^Sr ratios, as indicated by the longer whisker tails towards more radiogenic ratios. The box-and-whisker plots reveal three statistical outliers (one from the *streams+lakes* group and two from the *plants* group). While we aim at calculating baselines on a regional scale, we exclude these statistical outliers, expecting them to represent local anomalies. This is to avoid such values having a disproportionate effect on the means calculated for larger regions. The assembly of all proxy data from samples across Scania computed in this study define a mean of ^87^Sr/^86^Sr 0.7133 ± 0.0030 (1*σ*; n = 102), and with a range from 0.7082 to 0.7216 (Table 3). Grouping of the data based on proxy-type results in the following statistics: surface waters (*streams + lakes*) define the lowest Sr isotope ratio mean value with ^87^Sr/^86^Sr = 0.7129 ± 0.0023 (1σ; n = 36); followed by soil leachates with mean ^87^Sr/^86^Sr = 0.7132 ± 0.0041 (1σ; n = 24); and finally by plants with the highest mean value of ^87^Sr/^86^Sr ratios = 0.7138 ± 0.0030 (1σ; n = 42) (Table 3). Soil leachate data indicates highest variability in their ^87^Sr/^86^Sr signatures, as reflected by the elevated standard deviations and larger data range of this proxy archive, followed by plants, then surface water (*streams + lakes*) which show the lowest variability (Table 3). We note a distinct proxy archive data overlap in our biosphere data set clearly depicted in the box-and-whisker plot (Fig 6).

**Table 3:** Descriptive statistics of bioavailable ^87^Sr/^86^Sr ratios from Scania.

	*Scania-wide baseline statistics*			
* *	All	Streams+lakes	Soil leachates	Plants (leaves)
Mean	0.7133	0.7129	0.7132	0.7138
Standard error	0.0003	0.0004	0.0008	0.0005
Median	0.7129	0.7126	0.7120	0.7136
Standard deviation	0.0030	0.0023	0.0041	0.0030
Range	0.0134	0.0089	0.0150	0.0116
Minimum	0.7082	0.7091	0.7082	0.7092
Maximum	0.7216	0.7180	0.7232	0.7208
Number	102	36	24	42
Confidence (95.0%)	0.00058	0.00079	0.00171	0.00093

Outliers are identified and removed before calculations, see text for details.

In the following section, we explore regional patterns observed with respect to the distributions of strontium isotope ratios for the analysed proxies. We focus on the inter-site variation between proxies, whereas the variation between proxies at specific sites (intra-site proxy variation) is presented in detail in the supporting information.

#### Controls on bioavailable strontium isotopic ratios by bedrock lithology

Fig 5 presents the multi-proxy Sr isotope signatures from individual sampling sites of the present study, grouped by the lithology of the underlying bedrock. Here, we discriminate between sites located within areas dominated by crystalline magmatic and metamorphic rocks (Precambrian in age), which are grouped together in the granites/gneisses group, and sites within areas dominated by younger, pre-Quaternary sedimentary rocks, which are separated in sub-groups dependent on bedrock type. Considerable data overlap exists between the lithological groups, with proxy data from granites/gneiss and marls being the only groups distinguishable from each other. Data from different proxies within each lithological unit exhibit considerable variation in ^87^Sr/^86^Sr ranges, with multi-proxy archive data from the mud-, clay-, and siltstone group being the most homogenous with respect to the ranges defined by the proxies. Multi-proxy data from sites dominated by marl bedrock have a range of strontium isotopic ratios distinctly lower than the rest of samples, with the exception of the soil sample from site 4. However, we must note that lower variation of the data could be due to a low number of sampling sites that meet this category. Within the sandstone group, data from the majority of northernmost sites within the Cretaceous sandstones of the Kristianstad area (sites sb-32, sb-6, sb-3, sb-4) and of the centrally located Jurassic sandstone (sites 16, 16a, 11 and 11a) show a tendency to have elevated radiogenic strontium isotope signatures compared to samples from other, geographically distinct sandstone dominated areas (Paleozoic sandstone: sites sb-13, sb-27 and 8; Jurassic and Triassic sandstone: sites 21 and 20), but also from the coastal sites of sb-33 and sb-34 (Cretaceous sandstone) (Table 2 and Fig 3). This indicates, that the sandstone group of proxy data could be further divided into subgroups, but these groups would still show overlapping ranges, and would not define geographically discernible entities (Fig 3). The high variability of strontium isotope ratios in the areas dominated by gneiss, granites, and clastic sedimentary rock expected due to the nature of their composition, which has been shown in several other studies [36,62–64]. Despite a comparatively narrow range of proxy-data from limestone dominated areas, the bioavailable Sr isotope signatures from this group largely exceeds the range expected for Cretaceous–to Tertiary limestone-derived Sr components (^87^Sr/^86^Sr ~0.708–0.709; [65]), especially those signatures from the northern sites within the Cretaceous limestone of the Kristianstad area (Sites sb-10, sb-11, sb-18, sb-24, sb-30, sb-31 and sb-35; Table 2; Figs 3 and 5). This is likely due to an admixture of radiogenic Sr components from clastic gneiss-granite components in glaciogenic sediments overlying the limestones. Finally, none of the lithological groups are spatially coherent and each group holds bedrock spanning several geological ages (Table 2), which could also contribute to the lack of distinguishable strontium isotope intervals. In conclusion, grouping proxy data in groups according to underlying bedrock lithology does not seem to give discernible entities of bioavailable strontium ratios.

#### Controls on bioavailable strontium isotopic ratios by coastal proximity

Coastal proximity can influence bioavailable Sr isotope signatures through addition of sea spray carrying strontium with isotopic ratios close to that of modern seawater (~^87^Sr/^86^Sr = 0.7092 [65]). This could be an important feature in our study, as shown in studies by [66–68]. Although the highest concentrations of sea spray is found within 10–20 km from the coast [66], potential deposition can be spread as far as 300 km inland. Sampling sites located within 1 km of the nearest coast are identified and marked with a rectangle (Fig 5). Within the Precambrian group, two coastal sites (sb-25 and sb-26), along with site sb-12 located further inland, have markedly lower strontium isotopic ratios than other sites within the Precambrian bedrock dominated areas, which we could interpret to reflect addition of unradiogenic Sr via sea spray to these respective soils. However, it is important to note that these three sites are from within small Precambrian outcrop windows within areas otherwise dominated by Paleozoic sediments, and potentially this proximity to younger bedrock could also influence the bioavailable strontium ratios of the respective proxies (Fig 3). From the limestone group, situated in a southern area dominated by Cenozoic sediments, measured ^87^Sr/^86^Sr ratios from site 23 show elevated strontium isotopic ratios compared to site 1, which is situated further inland within the same lithology (Table 2; Fig 3), and thus site 23 seems not to be dominated by sea spray carrying Sr isotopes with ratios ^87^Sr/^86^Sr ~0.7092. Site 23 is located downstream from areas dominated by different lithologies (Fig 3), which could be one reason for the observed discrepancy, and for making strontium isotopic ratios of site 23 similar to sites 4, 4a and 4b. Elevated strontium isotope signatures of samples from the remaining sites within the Limestone group could be caused and controlled by the location of these sites near the northern Precambrian terranes, as discussed in previous section. Within the sandstone group, the lowest strontium isotope ratios are located in the coastal areas, with site sb-16 as a notable exception. While this indicate an influence by sea-spray at these sites, the distinct sandstone outcrops across the region, spanning in age from Paleozoic to the Mesozoic, could hold a large, inter-site Sr isotopic variation, depending on the difference of the sediments they originally formed from. Within the shales group, the coastal sites (site 6 and 7) show the lowest strontium isotopic ratios, perhaps indicating the influence of sea spray, while the most radiogenic Sr isotopic ratios are located in central Scania (sites 10 and 12), perhaps indicating an influence from the Precambrian terranes. However, due to the large variation of the strontium isotopic ratios within the group, and the low number of sampled sites, it is not possible to conclude if this is a general trend. The variation of the coastal sites across the bedrock groups is too high to set apart the coastal sites as a single group. Despite the tendency of some coastal sites to show lowered ^87^Sr/^86^Sr ratios than other sites within their bedrock group, we are unable to constrain the different controls on the strontium isotopic signatures of these particular sites. This is mainly due to the presence of highly variable geological backgrounds at the sites. Overall, we we determine that sea-spray related Sr contribution to the biosphere fractions studied herein do not explain regional baselines’ characteristics and compositional ranges.

#### Controls on bioavailable strontium isotopic ratios by geographical distribution of bedrock outcrops

We sorted our data according to latitude of the sites within the respective lithological groups (Fig). This was due to 1) general NW-SE directed outcrop patterns of the different terranes in Scania (Fig 2), and 2) the basement and overlying bedrock (sediments) becoming younger in a NE-SE direction. We are able to demonstrate a general increase in ^87^Sr/^86^Sr ratios with respect to latitude in each bedrock type group, however sites sb-33, sb-34, 20 and 21 within the sandstone group are notable exceptions (Fig 5). A positive relationship between bioavailable Sr isotope ratios and latitude was observed. This could be due to an increasing relative contribution of bioavailable Sr from Precambrian bedrock relative to a less pronounced contribution from overlying sedimentary bedrock, whose outcrops and respective sediment thicknesses decrease from south to north. Therefore, the observed data appear to reflect the proximity of the sampling sites to areas dominated by Precambrian bedrock.

#### Definition of spatially coherent areas with similar strontium isotopic signatures

**Fig 7:**
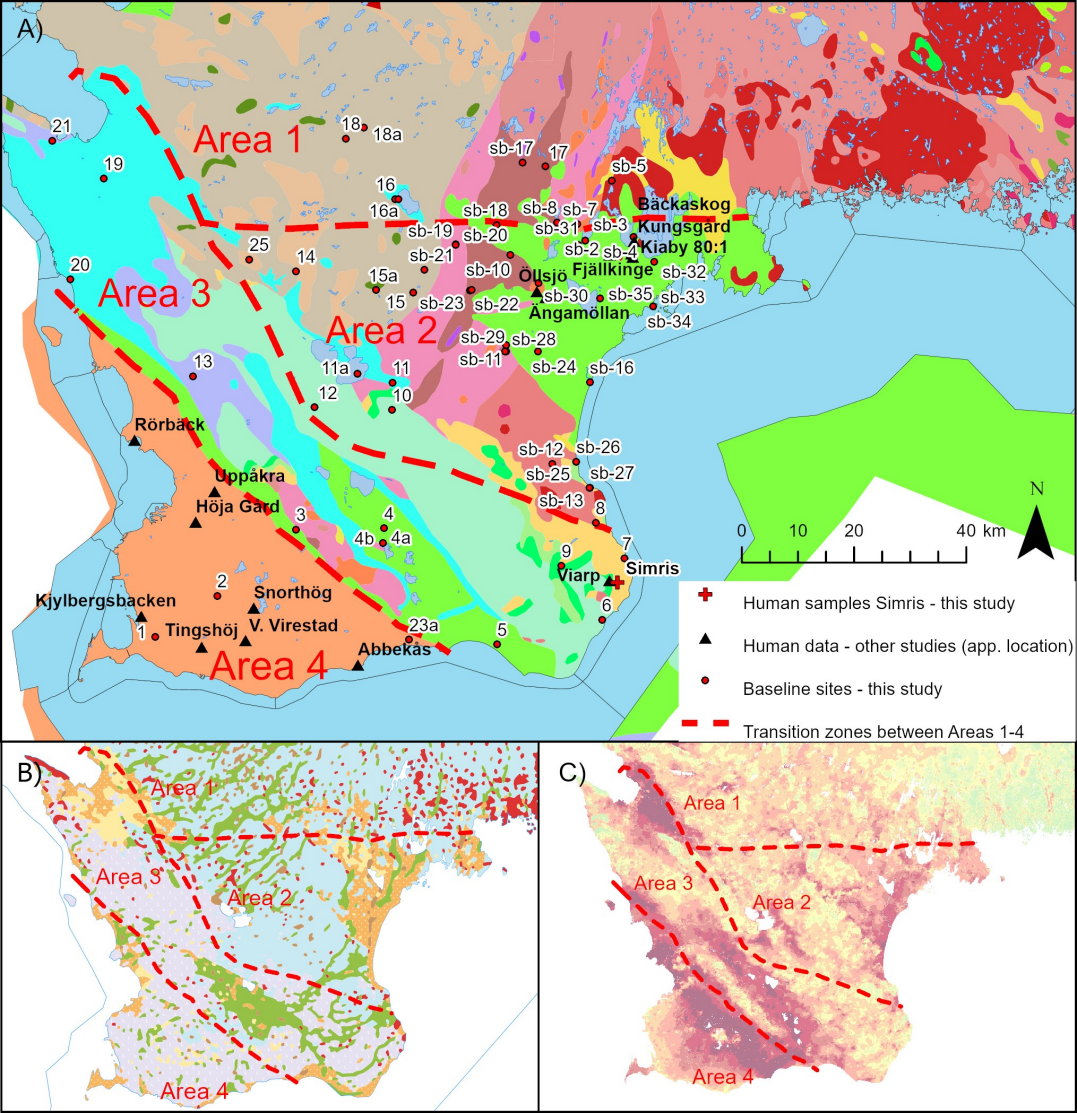
Maps of the three sub-areas (Areas 1, 2 and 3–4) of the Scania region for which individual multi-proxy baselines are presented. Border zones should be interpreted as transition zones, illustrating the gradual nature of the transition between zones characterised by different Sr isotope ranges. Maps show approximate site locations of excavation sites of humans from Late Neolithic and Early Bronze Age sites (Bäckaskog Kungsgård, Kiaby, Kiaby Mosse, Öllsjö, Ängamöllan, Viarp, Rörbäck, Höja Gård, Kjyllbergsbacken, Snorthög, Tingshøj, V.Virestad and Abbekås [13], Iron Age site (Uppåkra [15]), and Late Bronze Age site from Simris II (this study). References and legends of thematic maps shown on Figs 3 and 4.

**Fig 8:**
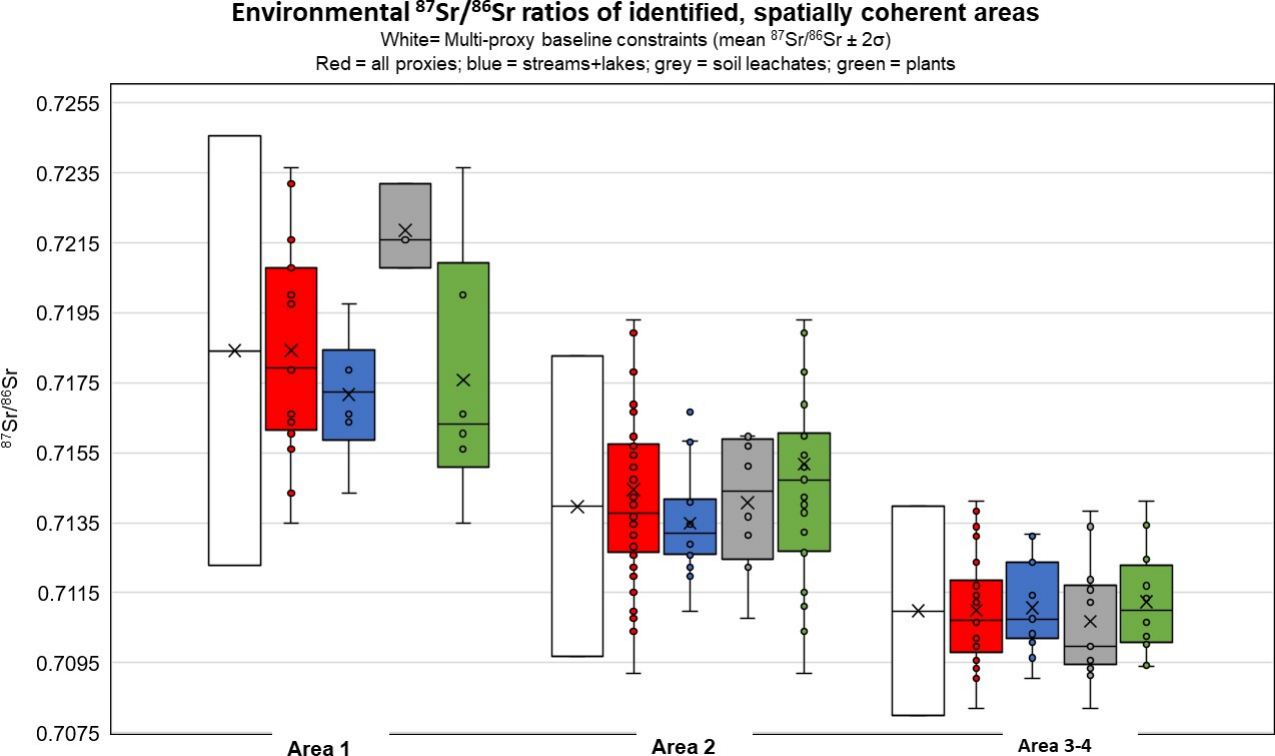
Box-and-whisker plots of the strontium isotope ratios of the different environmental proxies divided into the three sub-areas of Scania. Areas are defined by their isotopic range in combination with the respective geographical position of the sampling sites (Table 2; Fig 7). Multi-proxy baseline constraints are calculated as mean 87Sr/86Sr ±2σ). The statistical outlier from Area 2 (plant from site sb-22; ^87^Sr/^86^Sr = 0.7374) is not shown on plot.

In Scania, bedrock lithology and coastal proximity are unsuitable criteria to define meaningful groups with geographical coherence in their biosphere ^87^Sr/^86^Sr data. Hence, they could not serve as basis for the definition of respective baselines. We did, however, observe a general increase in ^87^Sr/^86^Sr ratios of the sites with respect to latitude in each bedrock type group. In turn, we structured our data by taking the geographical location and the displayed pattern of bioavailable strontium isotope signatures into consideration. In the following we defined four sub-regions within Scania as a consequence of this. The sites of the northern Precambrian areas show the highest bioavailable ^87^Sr/^86^Sr ratios, and are separated into Area 1, covering approximately 2400 km^2^, with a spatial sampling density of approximately 1 site pr. 270 km^2^ (9 sampling sites; 16 multi-proxy samples) (Fig 7). Area 2 comprises sites that are located on a variety of bedrock lithologies, ranging from Mesozoic sand- and limestone dominated terranes in north-eastern Scania, to sites within Precambrian terranes in the south, characterised by ^87^Sr/^86^Sr ratios resembling those measured in proxy archives from the aforementioned terrane (Fig 7). We observe elevated isotopic ratios compared to the other sites in the horst and graben landscape in centrally located sites 10 and 12 (Paleozoic shale) and sites 11 and 11a (Mesozoic sandstone). These are added into Area 2, together with the coastal sites 8, sb-27, sb-13 (Paleozoic sandstone) and sb-12, sb-25 and sb-26 (Precambrian granodioritic gneiss), to outline an area with geographical coherence and similar ^87^Sr/^86^Sr ratios. Area 2 covers approximately 2900 km^2^, giving a spatial sampling density of approximately 1 sampling site pr. 100 km^2^ (29 sampling sites; 40 multi proxy samples) (Fig 7). Area 3, covering approximately 3400 km^2^, comprises the remaining samples from the NW-SE oriented horst and graben landscape (Fig 7). Area 4, covering approximately 1400 km^2^, comprises the sediments of Cenozoic age, with the borders of this area characterised by geological lineaments (Fig 7). Only sites 1, 2 and 23 lie within this area, and proxy-archive strontium isotopic signatures are similar to those of Area 3. For this reason, we merged Area 3 and 4 into one geographical entity, covering approximately 4700 km^2^, with a spatial sampling density of approximately 1 site pr. 310 km^2^ (15 sampling sites; 39 multi-proxy samples). The grouped data are plotted as box-and-whiskers diagrams alongside multi-proxy baseline constraints of each group (Fig 8). A thorough discussion of the Sr isotope distributions in the Scania-wide and sub-regional multi-proxy baselines is given in the supporting information, where the measured environmental data is depicted alongside calculated baselines in S2 and S3 Figs.

The statistics of each areal group, after removal of statistical outliers, are given in Table 4. As shown very clearly in the box-and-whisker diagrams (Fig 8), the proxies within Area 1 define very different mean values and distributions. Soil leachate and surface water Sr isotope signatures were observed to differ from each other and from the wider ranging plant signatures. At this stage, since we are unable to precisely explain these discrepancies, we emphasise that this difference could be an effect of low sampling densities, especially from the streams, lakes and soil leachate groups within this particular area. Also, the spread in ^87^Sr/^86^Sr signatures in Area 1 indicates a complex mixing of Sr from different sources, particularly from the significantly different bedrock in this Precambrian area, which are distributed in the area non-uniformly and spanning a wide range of geological ages, and causing an enhanced heterogeneity in the proxy archive data (Figs 8 and S3). Further, these discrepancies support our suggestion to apply the multi-proxy baseline of Area 1 (and similar terranes) with caution, as even more caution should be taken if relying on single-proxy data. Therefore, more detailed sampling of different proxies is necessary to better constrain the baseline of this northern area in Scania, and additional baseline samples from archaeological sites should be included in future studies on mobility within these areas. The multi-proxy baseline of Area 1 (mean ^87^Sr/^86^Sr = 0.7184 ± 0.0061 (2σ; n = 16)) is compliant, however still somewhat lower, with the baseline defined from areas of crystalline, Precambrian terrane surrounding the area of Falbygden (Fig 1), which has a reported baseline defined by mean ^87^Sr/^86^Sr = 0.7220 ±0.0032 (1σ; n = 39) [28]. From Mälardalen within the Precambrian Swecokarelina orogen (Fig 1) the reported baseline of mean ^87^Sr/^86^Sr = 0.7335 ± 0.0070 (1σ; n = 4) is higher compared to Area 1 [31]. Looking into other studies from Europe, areas with similar, albeit not entirely identical, strontium isotope ranges as compared to Area 1 are reported from areas within the massifs of France, as well as areas within the Vosges, the Pyrenees, the western part of the Iberian Peninsula, parts of the Italian and Austrian Alps, as well as smaller parts of northern England, Ireland and eastern Europe [36,58,64,68,69].

**Table 4:** Descriptive statistics of bioavailable ^87^Sr/^86^Sr ratios from Scania.

** **	***Area 1 baseline statistics***			
*** ***	**All**	**Streams+lakes**	**Soil leachates**	**Plants (leaves)**
Mean	0.71843	0.71716	0.72186	0.71804
Standard error	0.00077	0.00075	0.00071	0.00134
Median	0.71793	0.71724	0.72159	0.71662
Standard deviation	0.00307	0.00183	0.00123	0.00355
Range	0.01016	0.00541	0.00242	0.01016
Minimum	0.71350	0.71435	0.72078	0.71350
Maximum	0.72366	0.71976	0.72320	0.72366
Number	16	6	3	7
Confidence (95.0%)	0.00164	0.00192	0.00306	0.00328
	***Area 2 baseline statistics***			
	**All**	**Streams+lakes**	**Soil leachates**	**Plants (leaves)**
Mean	0.71397	0.71328	0.71407	0.71425
Standard error	0.00031	0.00034	0.00069	0.00052
Median	0.71373	0.71293	0.71439	0.71448
Standard deviation	0.00215	0.00132	0.00194	0.00255
Range	0.01012	0.00487	0.00523	0.01012
Minimum	0.70918	0.71096	0.71076	0.70918
Maximum	0.71930	0.71584	0.71598	0.71930
Number	48	15	8	24
Confidence (95.0%)	0.00062	0.00073	0.00162	0.00108
	***Area 3+4 baseline statistics***			
	**All**	**Streams+lakes**	**Soil leachates**	**Plants (leaves)**
Mean	0.71098	0.71107	0.71068	0.71124
Standard error	0.00024	0.00033	0.00047	0.00044
Median	0.71066	0.71075	0.70997	0.71100
Standard deviation	0.00149	0.00128	0.00169	0.00152
Range	0.00591	0.00411	0.00562	0.00473
Minimum	0.70820	0.70905	0.70820	0.70939
Maximum	0.71412	0.71317	0.71383	0.71412
Number	39	15	13	12
Confidence (95.0%)	0.00048	0.00071	0.00102	0.00097

Statistics of sub-regional areas within Scania, for which the baselines are applicable to studies of prehistoric mobility. Delineation of areas is based on a combination of geography, bedrock geology, and ^87^Sr/^86^Sr ratios of the proxies. Outliers are identified and removed before calculations, see text for details.

Area 2 is more uniform with respect to mean values of Sr isotope ratios for the different proxies, however, the individual proxies still have different data ranges (Table 4; Figs 8 and S3). In both Area 1 and Area 2, the plant ^87^Sr/^86^Sr values exhibit the largest ranges, and consequently define the largest standard deviations in their distributions. However, all calculated (mean ^87^Sr/^86^Sr ± 2σ) single-proxy baselines of Area 2 are consistent and compatible with each other, yet with varying scatter (S3 Fig). This is not the case for Area 1. The calculated, single-proxy water baseline of Area 2 is the narrowest baseline, with a range identical to the ranges defined by the measured data. However, single-proxy baseline ranges calculated for plants and soil leachates baseline exceed the majority of the measured data points within each of the proxy archives, for the soil leachates towards both the upper and lower constraints, and for the plants notably towards to lower constraint (S3 Fig). From this, it is advisable to use multi-proxy data to constrain the baseline of Area 2 to mean ^87^Sr/^86^Sr = 0.7140 ± 0.0043 (2σ, n = 48) as a conservative choice, and additionally, site-specific baseline samples from archaeological sites should be included in future studies on mobility within these areas. The multi-proxy baseline of Area 2 is compliant to the baseline of mean ^87^Sr/^86^Sr = 0.7146 ±0.0014 (1σ; n = 45; water and fauna) reported from areas of similar geologies in the Falbygden area (Paleozoic, sedimentary bedrocks; Fig 1) [28]; and to the baseline of mean ^87^Sr/^86^Sr = 0.7140 ±0.0024 (1σ; n = 25; fauna) reported from the island of Öland (Paleozoic limestone; Fig 1) [31]. From the island of Gotland, also dominated by Paleozoic, sedimentary bedrock, the reported baseline is still compliant, however somewhat lower (mean ^87^Sr/^86^Sr = 0.7120 ± 0.0018 (1σ; n = 26; soil and fauna) [31,32]. While these baselines are defined by variable proxies, this supports our approach for a construction of baselines that rely on multi-proxy data to best cover the biosphere ranges of the regions. Area 3–4 is characterised by uniform bioavailable Sr isotope signatures, as expressed by the defined statistics of the different proxies (Table 4). Subsequently, these areas are Scania’s most homogeneous regions with respect to biosphere Sr data in this study. Only the soil leachate baseline shows a calculated lower limit largely exceeding the measured data, indicating that a single-proxy baseline relying on only soil leachates, is not adequate enough to constrain a reliable baseline with the sampling density of the present study (Table 4 and S3 Fig). The baseline of Area 3–4 is characterised by the least radiogenic (lowest baseline constraint of all areas), which is likely to reflect the increased content of limestone-derived Sr from either the bedrock themselves, and/or from increased clastic carbonate components in the overlying tills in this area. The comparatively good agreement of individual proxy baselines calculated for Areas 3–4 compared to Areas 1 and 2 (Figs 8 and S3) possibly lies in the nature of the bedrock in this area. The predominantly calcareous sediments underlying the clayey, carbonate rich Baltic tills are a strong buffering system for Sr and its isotope composition against the radiogenic biosphere components that derive from relatively Sr-poor bedrock and sandy tills that prevail in the other areas. This is a pattern also seen in a recent study in Cyprus [37], where single-proxy baseline differences were minimal in carbonate bedrock terranes, and more pronounced in crystalline, ophiolite bedrock-dominated terranes. The multi-proxy baseline for Area 3+4 which is very much compatible with the baseline established for neighbouring country Denmark (excluding Bornholm), currently defined as ^87^Sr/^86^Sr = 0.7096 ± 0.0016 (2σ) [60]. Also, the baseline established for the Netherlands using archaeological fauna [70] is compatible with the strontium isotopic range of Area 3–4. Similar ranges are commonly found within the sedimentary basins of Europe, both in site-specific studies in e.g. Germany, Hungary and Italy to mention a few [48,57,71] and in broader studies, e.g. [36,58,68,69].

The outlined areas (Area 1, 2 and 3–4), which we defined based on Sr isotope signatures, do not coincide fully with lithological terranes (Fig 7). However, when comparing the data-defined areas with the distributional patterns of surface soil types (Fig 7B), it becomes evident that borders between Areas 3, Area 1 and 2 coincide with zones characterised by changes in soil compositions. Areas 1 and 2 are dominated by sandy moraines overlying and/or in proximity to Precambrian gneiss and granite areas, whereas Area 3–4 is characterised by clayey moraines above predominantly Paleozoic, Mesozoic and Cenozic sedimentary deposits. As mentioned earlier, the glaciogenic tills of Scania were deposited from ice lobes transporting debris from areas to the north and northeast, and from ice lobes moving in from the south. Thus, the difference in biosphere strontium isotopic ratios between Areas 1, 2 and Area 3–4 could be partially controlled by differences in the debris sources, and in turn by glaciation history. While the tills in Area 1 and 2 are likely dominated by material from Precambrian crystalline terranes, tills of Area 3–4 are likely dominated by material transported into Scania from areas in which carbonate-rich sedimentary rocks of the Baltic prevail. Although glacier transported material can be derived from up to several 100 km away, large volumes of clastic materials in tills have been identified to be of local origin [28,72]. This supports our tentative interpretation. Similar to Denmark [60,73], it is reasonable to assume that the biosphere Sr signatures of Scania are dominated by both bedrock (Precambrian basement rocks and Paleozoic, Mesozoic and Cenozoic sedimentary rocks), and by Sr derived from Quaternary glaciogenic deposits. This mixing of Sr components appears to be responsible for the blurring of lithological border zones with respect to biosphere Sr signatures, and most likely to be expected in riverine systems that pass through different terranes. This blurring effect on biosphere Sr was also recently described in a study of the Falbygden area of central Sweden [28,35].

Overall, the expanded Sr isotope ranges observed in the multi-proxy data from Scania reflect the complex geology circumstances, dominated by (1) abrupt terrane boundaries along tectonic lineaments, (2) variety of juxtaposed lithological terranes, and (3) a diversified, often local effect of till and moraine landscapes superimposed on the bedrock that contribute to a complex multi-source mixing of bioavailable Sr in Scania.

### The Bronze Age site of Simris II in the light of the new Scania baselines

**Fig 9:**
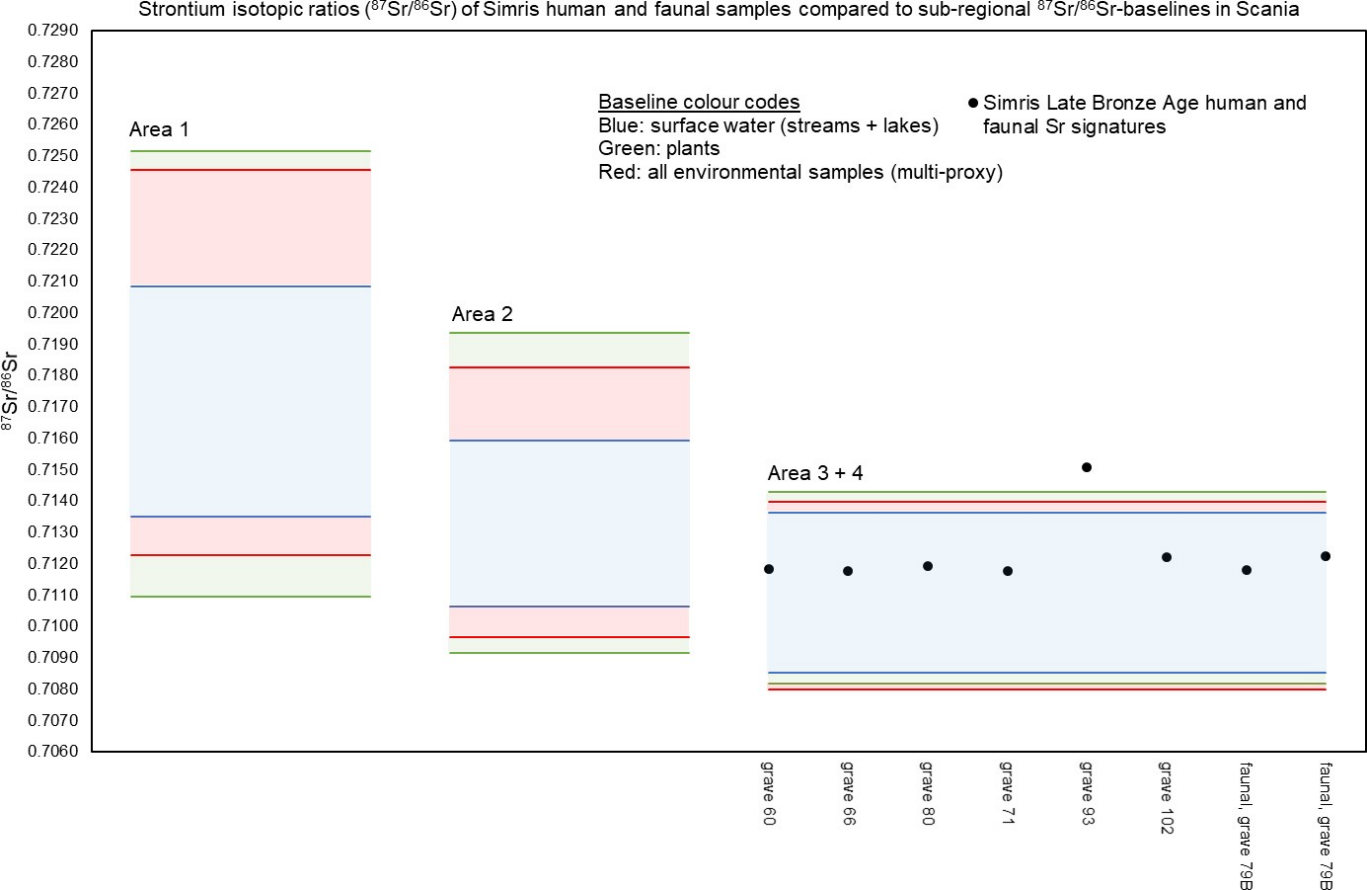
Strontium isotopic ratios of cremated human and faunal samples from the Late Bronze Age cemetery Simris II, shown together with sub-regional, multi- and single-proxy baselines from Scania. The Simris II cemetery is located within Area 3–4 (Fig 6). Results and details of the samples are found in Table 4.

**Fig 10:**
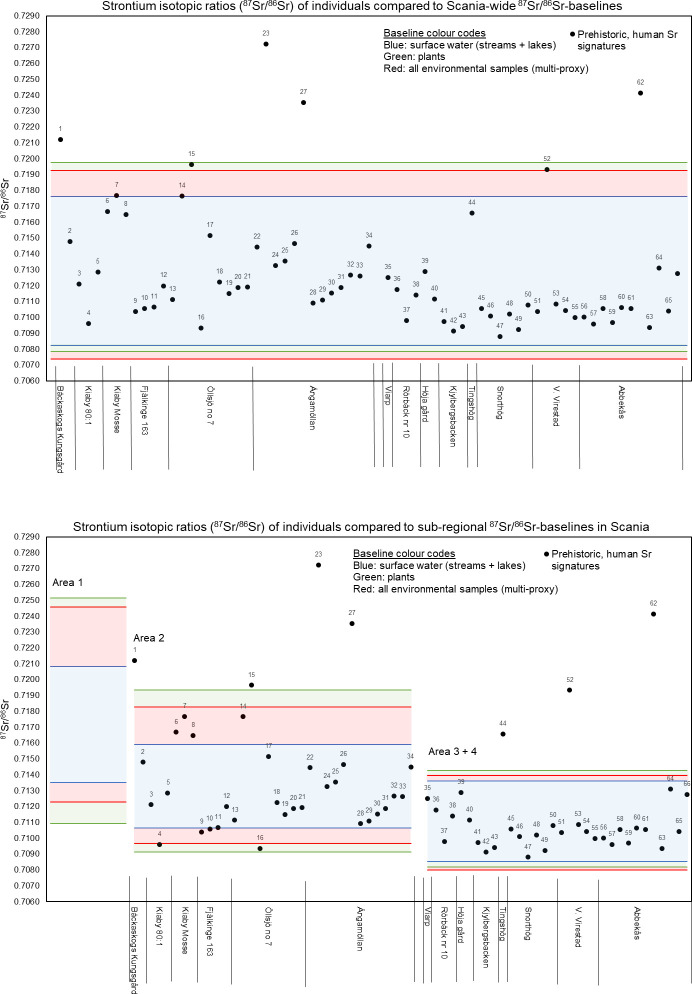
A. Prehistoric human Sr isotope signatures reported in [13], compared to Scania-wide baselines defined by multi-proxy and single-proxy archive data presented herein. Baselines are calculated as means of the measured strontium isotopic range of relevant proxies ± 2σ. Numbering of samples is successive to ease referencing in text and does not correspond to the original sample labels used in original publication. Five individuals fall outside the Scania-wide multi-proxy baseline using four digit ratios. B. Spatially categorised prehistoric human Sr isotope signatures reported in [13], compared to sub-regional baselines in Scania defined by multi-proxy and single-proxy archive data presented herein. Baselines are calculated as means of the measured strontium isotopic range of relevant proxies ± 2σ. Numbering of samples is successive to ease referencing in text and does not correspond to the original sample labels used in original publication. While five individuals have Sr isotope signatures outside the Scania-wide multi-proxy baseline (Fig 10A), nine individuals fall outside the sub-regional multi-proxy baselines proposed herein, using ratios with four decimals. No sites lie within Area 1.

The results of the strontium isotope analyses of six individuals and two fauna samples from Simris II are listed in Table 1, and are depicted on the diagram of Fig 10 together with the defined baselines of the Scania region. The fauna samples, which are possibly from lamb, fall within the local baseline, as defined herein by the Area 3–4. The human samples, except for one, exhibit very similar ^87^Sr/^86^Sr values and also fall within the local baseline (Fig 9). The individual from grave 93 falls outside the local baseline(s), which suggests an origin, or the place where early childhood was spent, outside the Simris II area. Rather, its Sr isotope signature is compatible with that from Areas 1 and 2, or from other areas outside Scania that are characterised by elevated Sr isotope signatures of the biosphere.

We selected the Simris II cemetery due to a number of factors: 1) the urns from grave 23 and 71 suggested acquaintances with long distance networks and exchange patterns; 2) a number of other graves were linked to stone monuments and furnished bronze items suggesting that the local community had a complex social stratification; 3) the site is located in an area of Scania showing a dynamic environment throughout the Late Bronze Age, thus likely involving various forms of mobility.

The number of human samples is too limited (6 out of 78 known individuals or c. 7.6% of the known buried population) to make statistics reliable; for all but one exhibits a narrow Sr isotope signature range hinting at their local origin within in Area 3–4, or at Simris II itself. For a methodologically correct interpretation of such data, we argue that one should take into account two critical issues: First, networking and mobility attested by archaeological evidence does not necessarily translate in human communities with high numbers of individuals of non-local origin by means of Sr isotope analyses. As discussed earlier, samples of petrous bone and, to a certain extent human enamel, provide information about the radiogenic characteristics of the landscape in which individuals spent their childhood. If no other comparative human bones and tissues from the same individuals are available, as in this case, it prevents any indications on when the individuals were mobile during their lives. Second, when an individual is cremated, it becomes feasible to consider an alternative scenario, whereby the remains could have been carried to a burial place, different from the original place of death. Although this was probably not a common practice, the possibility of a post-mortem mobility cannot be excluded [74,75].

Grave 93 contained the only individual of apparent non-local origin within our dataset. The archaeological context is slightly uncommon. In the first place it contained the cremated remains of two adult males (mixed in the same urn) [39]. While this is not an unknown practice, it is still unusual. The grave also contained a bronze razor with the handle in the shape of a bird’s neck and head of rare craftmanship and shape [39]. In a recent study, eleven sherds from ceramic vases found at Simris II, including the urn and lid from grave 93 have been analysed for their major and trace element compositions by means of Inductively Coupled Plasma-Mass Spectrometry (ICP-MS) by Sabatini et al. [47]. The samples belong to both urns and grave goods from eight different graves. The results of this study revealed that the urn from grave 93 and its lid were made of different clay and that the lid matched closely to the clay used to make the door urn from grave 71 [47]. More analyses of this kind are necessary to better understand the significance of such differences. However, these differences suggest a complex interplay between the chosen clay, the shape of the urns, and the characteristics of the grave goods. It has been argued that Late Bronze Age burial urns at Simris II were manufactured with the aim to communicate manifold messages linked to the identity and/or the social status of the deceased buried in them or of the group/kin to which he/she belonged [47]. This is consistent with what has been observed in other parts of southern Scandinavia. In the case of one of the two individuals in grave 93, the above mentioned peculiarities suggest a form of social distinction which, was also associated to a non-local origin. Consequently, our results are somewhat unexpected, since they mostly point to local origin for individuals whose cremated remains were buried inside particular house urns. This new knowledge invites to reassess research questions regarding the mobility of individuals from this site, as well as questions regarding their identities and their role(s) in long-distance networks.

### Application of new baselines to existing archaeological studies of human provenance and mobility in Scania

We apply the newly defined areal multi-proxy baselines of Scania (using the respective 2σ error bands) to a number of case studies, and explore the effects of using the Scania-wide baselines (Fig 10A) *vs* the regional (i.e., sub-area constrained) baselines (Fig 10B) we propose in our study. The human data used for the interpretations are previously published results of strontium isotopic analyses done on late Neolithic and early Bronze Age reported by Bergerbrant and co-workers [13]. Locations of the sites are shown in Fig 7. Number labelling in Fig 10A and 10B of the individuals from these sites are made in such way to simplify referencing in the text, and the numbers do not relate to the original sample labels in the respective studies. A key to identifying the original samples is contained in S1 Table. Human data are grouped according to excavation site in order to relate them geographically to the regional baseline of the site. No attempts to re-interpret individual provenance and archaeological contexts are undertaken, since the aim is to solely exploit the effects and consequences when relating the published data sets to our new baselines proposed herein.

Using ratios with four decimals, five individuals (no 1, 15, 23, 27 and 62) fall outside the Scania-wide, multi-proxy baseline, and seven individuals (in addition to the above, no 7, 52) fall outside the surface water based baseline (Fig 10A). This is equivalent to approximately 8% falling outside the Scania-wide multi-proxy baseline, and 11% falling outside the Scania-wide surface water baseline, indicating that at least 8% of the analysed humans have an origin outside of Scania. This is a lower percentage than the preliminary results of the original study, which indicated approximately 22% of the humans to be non-locals to Scania (based on Table 4 of the paper [13]), however using a tentative baseline [13] When we set the human Sr isotope data in relation to the area-specific baselines defined herein, a different picture rises with respect to local-, and non-local specification of the human data from Scania. Using ratios with four decimals, nine individuals (no 1, 4, 15, 16, 23, 27, 44, 52 and 62) fall outside the multi-proxy baseline, and five more (in addition no 6, 7, 8, 9, 14), if respective surface water areal baselines are considered. This is equivalent to approximately 14% of individuals falling outside the sub-regional, multi-proxy baselines, and 21% falling outside the sub-regional, surface water baselines. Out of these, only one human sample (no 23) shows a strontium isotopic ratio falling outside *all* sub-regional baselines defined herein, equivalent to 1.5% of the analysed humans having a provenance outside of Scania. Thus, the use of sub-regional baselines suggests that at least 14% of the analysed humans have originated from an area outside their burial place. However, all but one individual (no 23) could originate from an area within the Scania region (Fig 10B). This means that the identified mobility could be mainly regional within Scania, although, an origin of these individuals in other regions with similar strontium isotopic signatures cannot be excluded.

Due to the overlapping baseline ranges, many individuals have Sr isotope signatures compatible with the ranges of more than one area. This means that any non-locals of either area, falling within the range of ^87^Sr/^86^Sr signatures shared by the two areas, will not be identified using the Sr isotope methodology.

If only surface water baselines are considered, four individuals from Area 2 (no 6, 7, 8 and 14) are only compatible with an origin in Area 1, while three individuals (no 4, 9 and 16) are only compatible with an origin in Area 3–4. This shows that because the surface water proxy archive baseline is narrower than the multi-proxy baseline, cases (e.g. individuals no 6, 7 and 8 from Kiaby Mosse in Area 2) falling outside the surface water baseline, but inside the multi-proxy baseline, can appear. While the multi-proxy baseline in this case represents a conservative and recommended choice, it is also evident from Fig 9B that the majority of measured, bioavailable strontium isotope ratios within Area 2 lie within the constraints of the surface water baseline. Therefore, it seems is desirable to include more site-specific baseline samples local to burial sites like this, when interpreting provenance. In general, the human data from Area 2 are more scattered than human data from Area 3–4, and in this regard is compatible with the increased scatter in respective proxy data from Area 2 (S3 Fig). As we stated earlier, this is likely a result from the heterogeneous bedrock geology that characterises this area (Fig 7). The consequence of this increased heterogeneity is that only very conservative distinctions between local and non-local humans can be done. As an example, the archaeological sites of Backaskogs Kungsgård, Kiaby 80:1, Kiaby Mosse and Fjällkinge are all localised in a narrow area within Area 2 (Fig 7), but the strontium isotope ratios of the analysed individuals from these sites form quite distinctive groups. However, they all fall within the range of the multi-proxy baseline of Area 2. The variations of human data observed in relatively narrow sub-areas of Area 2 implies that provenance studies within Area 2 need to be based on more local, site-specific baseline characterisations in the future. The archaeological contexts in such cases can be decisive for the interpretation of mobility.

A closer look at Area 3–4 seems to indicate that there are two groups of human strontium isotope signatures that are characteristic of this region. Data from the sites of Kjylbergsbacken, Tingshög, Snorthög, V. Virestad and Abbekås are characterised by lowered ^87^Sr/^86^Sr ratios compared to the signatures of individuals from the sites of Simris (Fig 10), Viarp, Rörback and Höja gård (Fig 10B). However, they are similar to the ^87^Sr/^86^Sr ratios of individuals interpreted as possibly local to Uppåkra from the study by Price [15]. This grouping roughly corresponds to a geographical sub-pattern of the burial site locations, with the former sites located either in or close to Area 3 (dominated by Paleozoic and Mesozoic sediments), and the later sites located in the south-west part of Area 4 (dominated by Cenozoic sediments) (Fig 7). At this stage we are unable to relate this pattern to a corresponding pattern reflected by our baseline data, which is possibly due to the low density of baseline sampling sites. However, we hypothesise that the human data might mirror a possible pattern that is related to the geology. As such, one would expect that the part of Area 4 bordering Area 3 to contain erosional material from the horst and graben dominated terrain characterised by predominantly Paleozoic and Mesozoic sediments with slightly elevated Sr isotope signatures. In contrast, the predominance of carbonate sediments in the southwest part of Area 4 would contribute less radiogenic bioavailable strontium, reflected by less radiogenic Sr isotope signatures in humans unearthed from sites in this area. This could indicate that Area 3 and 4 have distinct bioavailable strontium isotopic baselines, with Area 4 characterised by less radiogenic ^87^Sr/^86^Sr ratios than Area 3. It also suggests that the divide between the two areas is located further to the south-west than indicated in Fig 7. Our tentative interpretation could also be supported by the differences in thickness of soil horizons (Fig 7C). It is maybe possible that increased radiogenic Sr contribution to the biosphere from thicker soils having more abundant components derived from the tectonically active Area 3 could be responsible for this smearing of radiogenic signatures into Area 4. This needs to be investigated in detail at some later time.

Our study demonstrates the importance of regional baselines for provenance and migration studies. We also emphasize the importance of the inclusion of multi-proxy archives into baseline characterisation, as well as the importance of sufficiently high sampling densities within each region. If surface waters are considered to be contributing significantly to the Sr intake of humans in general, then one should include such sources in baseline definitions. This is particularly important in areas characterised by surface waters with high Sr concentrations, which are commonly due to contributions from carbonate rich bedrock and Quaternary glaciogenic sediments with abundant carbonate clasts. This inclusion should be done despite the possibility that riverine systems used as direct drinking water sources can carry Sr isotope signatures that exogenous, in contrast to the point source data obtained from plant and soil leachates. We propose, that a conservative approach should also be chosen when defining the error term of baselines, such as using double standard deviations to define upper and lower baseline limits as applied herein.

## Conclusions

This study presents the results of strontium isotope analyses of human remains (cremated) from the Bronze Age burial site of Simris II in Scania (southern Sweden), providing the first set of data from the beginning of the first millennium BCE of this region. The archaeological evidence at the site of Simris II upholds of a community well embedded in long distance networks, making this particular site interesting when investigating social dynamics and mobility within the first millennium BCE within southern Sweden. As the region of Scania is, in general, rich in archaeological remains from different prehistoric and historic periods, there is a great potential for conducting provenance studies by applying the strontium isotope system. We therefore present the first comprehensive environmentally based multi-proxy (surface water-, plant- and soil leachate) strontium isotope baselines for Scania. Consequently, the aim of the study is two folded. Firstly, to shed light on mobility within the first millennium BCE, and secondly to construct a comprehensive Sr bioavailable baseline of Scania. Based on our environmental samples we propose sub-regional Sr isotope bioavailable baseline suitable for ancient and modern provenance studies.

Our baseline data results reveal a general north-south decrease in biosphere Sr isotope signatures. This latitudinal change in ^87^Sr/^86^Sr values corresponds with changes in the geological background of Scania. While the influence of Sr from Precambrian granite-gneiss terranes decreases in Scania southwards, the influence of Paleozoic, Mesozoic and Cenozoic limestone and carbonate-rich clay and marl increases. Finally, there appears to be an increased influence of clastic carbonate components in overlying glaciogenic Quaternary tills towards the south-west. Consequently, we propose that rather than working with a Scania-wide multi-proxy baseline, which we define as ^87^Sr/^86^Sr = 0.7133 ± 0.0059 (n = 102, 2σ), multi-proxy baselines valid for three specific areas within Scania should be used in the future. These areas were defined by a combined geographic and Sr isotope classification, and took the geological background of the sites into consideration.

Our results show that the complex geology of Scania does not allow for a spatially meaningful, geology-based grouping of multi proxy data that could be beneficial for provenance studies. This is due to the highly complex and spatially scattered lithologies of the region, and a diversified, often local effect of till and moraine landscapes superimposed on the bedrock that contribute to a complex multi-source mixing of bioavailable Sr in Scania. Instead, a division into areas that are not fully corresponding a priori to the distribution of bedrock lithology is required. We define area specific, multi-proxy baselines are as follows: Area 1, farthest to the north, with ^87^Sr/^86^Sr = 0.7184 ± 0.0061 (n = 16, 2σ); Area 2, comprising the mid and western part of Scania, with ^87^Sr/^86^Sr = 0.7140 ± 0.0043 (n = 48, 2σ); Area 3–4, roughly corresponding to a northwest-southeast trending zone dominated by horst-graben tectonics across Scania, plus the carbonate dominated south- western part of Scania with ^87^Sr/^86^Sr = 0.7110 ± 0.0030 (n = 39, 2σ).

The complexity of the geology of Scania requires high spatial density sampling of multiple proxies to adequately constrain the baseline ranges, particularly of those areas dominated by Precambrian lithologies. Irregular sampling densities of different proxy archives could lead to single-proxy baselines not fully compliant with each other, as demonstrated for Area 1. The existence of differences in individual proxy baselines observed in Area 1, requires a refined approach when characterising baselines. The main reason for these differences is likely in the Sr-poor biosphere fractions produced in non-carbonate Precambrian crystalline terranes, which are vulnerable to very local and differentiated contributions of Sr (particularly from locally, isotopically inhomogeneous soil solutions). Here, the averaging effect of biosphere Sr in surface water might be beneficial for the characterisation of baselines. Hence, provenance and mobility studies in geologically complex regions like Scania require statistically sound sampling of all sub-areas and inclusions of all proxy archives.

Our area-specific baselines allow for a refined categorisation between local and non-local origin of human individuals unearthed from archaeological sites in Scania. The strontium isotope analyses of the human and faunal remains from Simris II suggests that most of the humans had a local origin, except for one individual. That individual could have originated from both Area 1 and 2 of Scania, regions north of Scania with a corresponding Sr baseline, or other regions with corresponding Sr baselines. In other words, all individuals investigated from Simris II could have originated within the Scania region. These results are somewhat unexpected, and raise new, important questions concerning our understanding of mobility and identity in Nordic Late Bronze Age communities.

Finally, we propose that for a methodologically correct interpretation of such data, two critical issues should be taken into account: 1) mobility between places characterised by similar bioavailable strontium baselines will not be revealed solely relying on Sr isotope data; 2) the possibility of a post-mortem mobility (by transfer of cremated remains) cannot be excluded, although this is likely not a common practice.

## Supporting information

S1 Fig Delta (Δ^87^Sr/^86^Sr) values of ^87^Sr/^86^Sr ratios of site specific pairs of proxies. (TIF)

S2 Fig Scania- wide baselines calculated from environmental proxies. (TIF)

S3 Fig Sub-regional baselines calculated from environmental proxies. (TIF)

S1 Table Key to identification of human samples from previous studies. (TIF)

S1 File (DOCX)
