# Supplementary figures and images for: Testing Late Bronze Age mobility in southern Sweden in the light of a new multi-proxy strontium isotope baseline of Scania
Source: PLoS One. 2021 Apr 21;16(4):e0250279. doi: 10.1371/journal.pone.0250279 (PMC8059841; doi:10.1371/journal.pone.0250279)

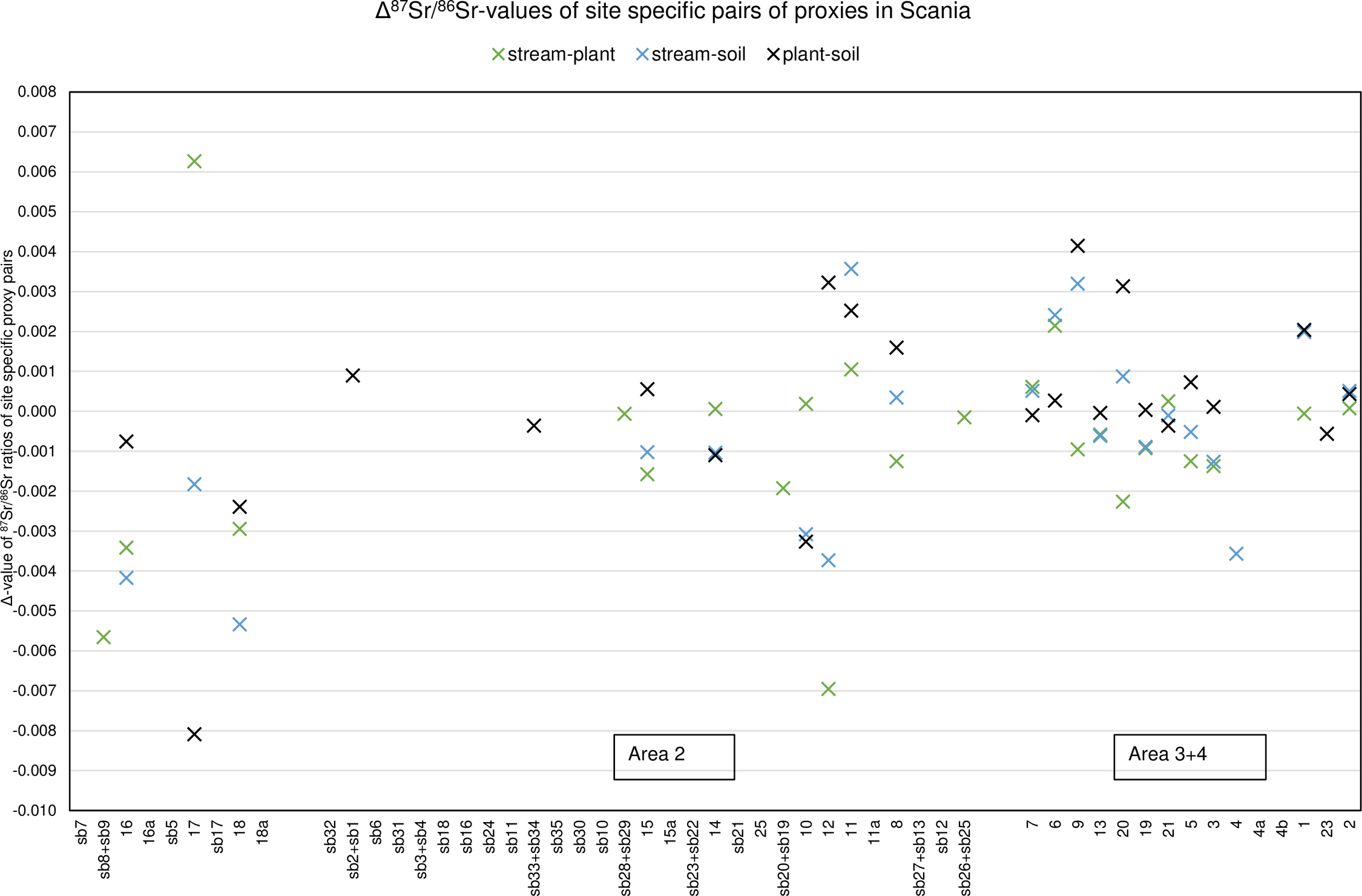

Supplement: S1 Fig — (TIF) [file pone.0250279.s001.tif]

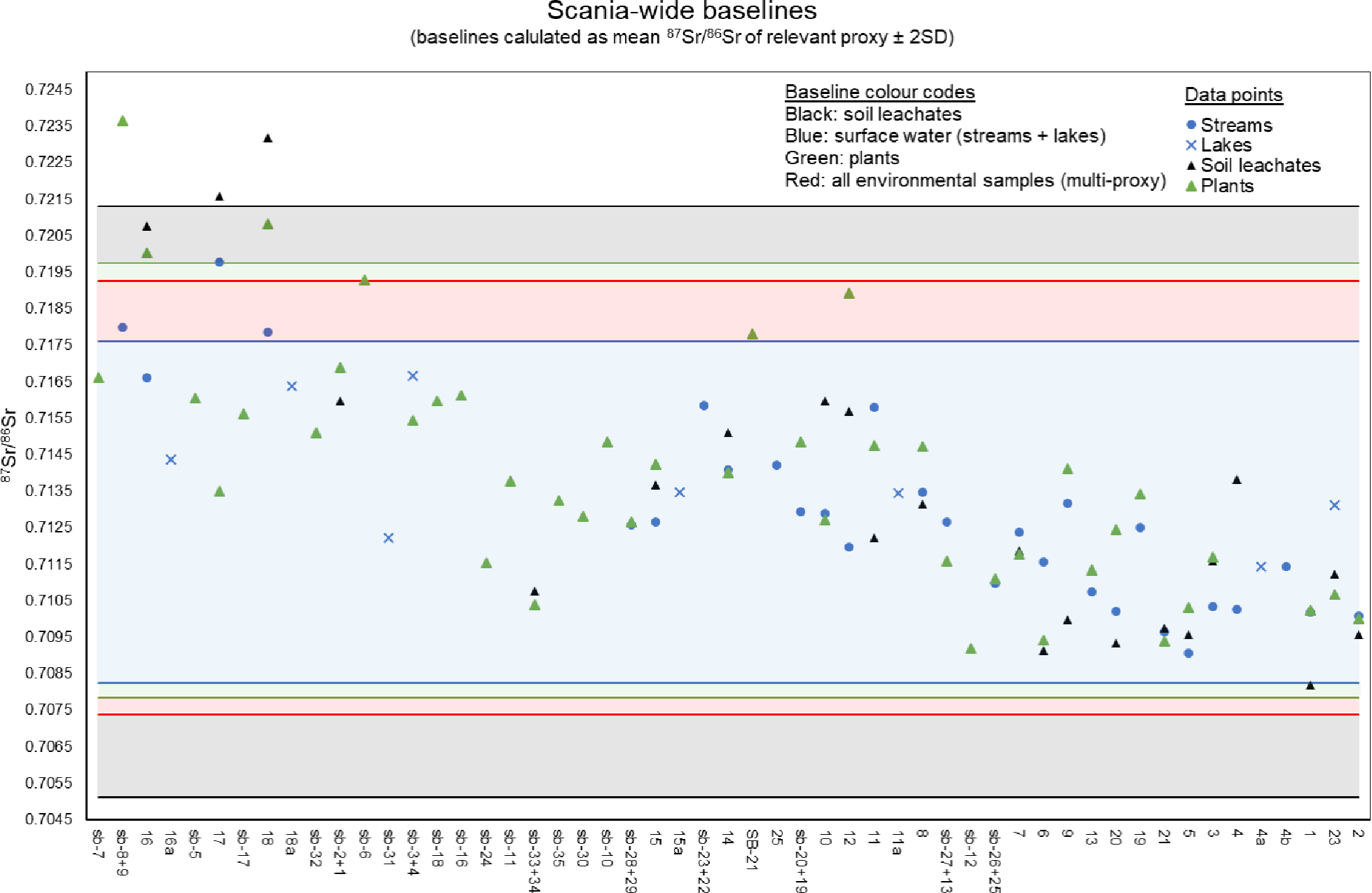

Supplement: S2 Fig — (TIF) [file pone.0250279.s002.tif]

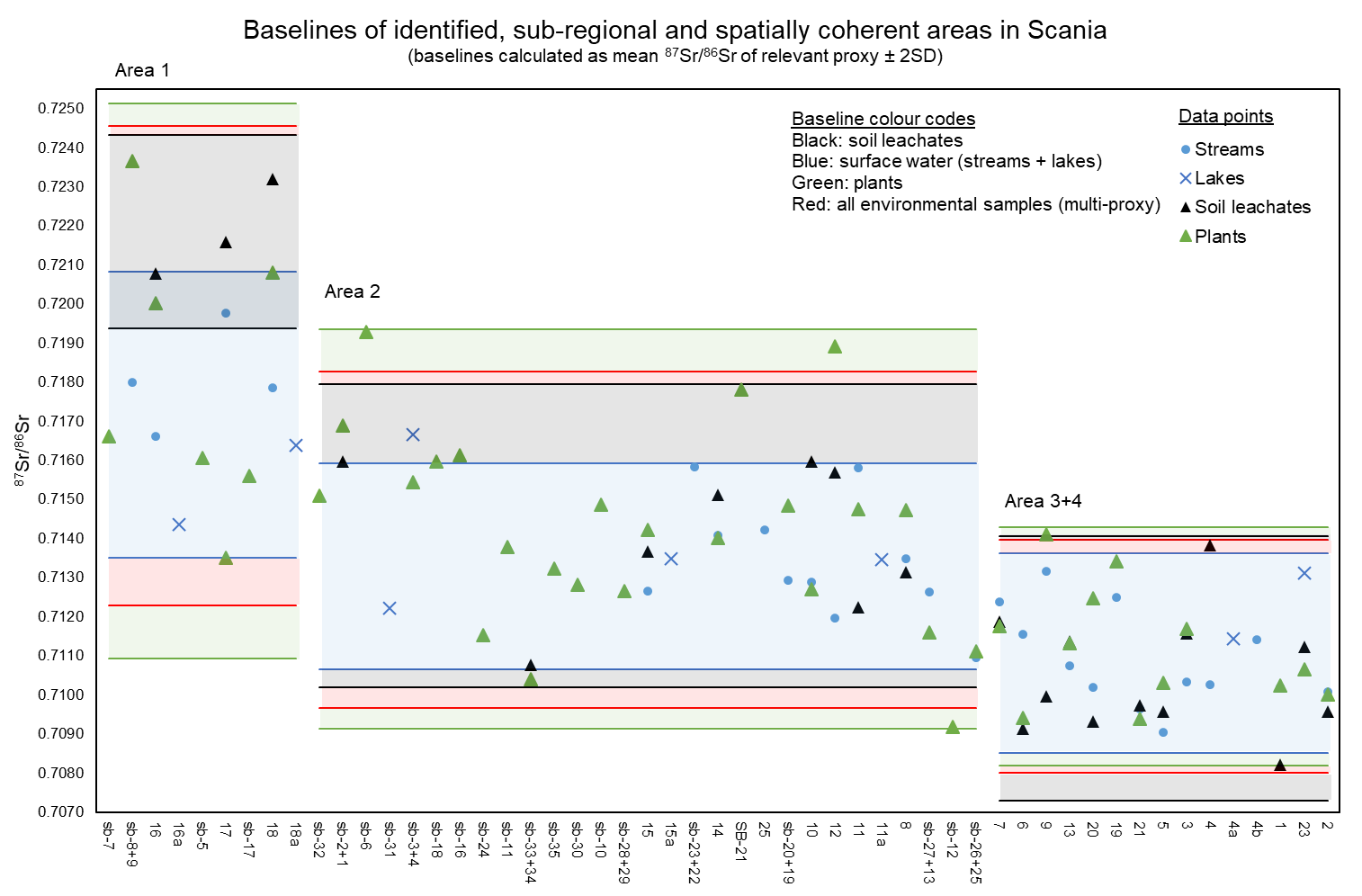

Supplement: S3 Fig — (TIF) [file pone.0250279.s003.tif]

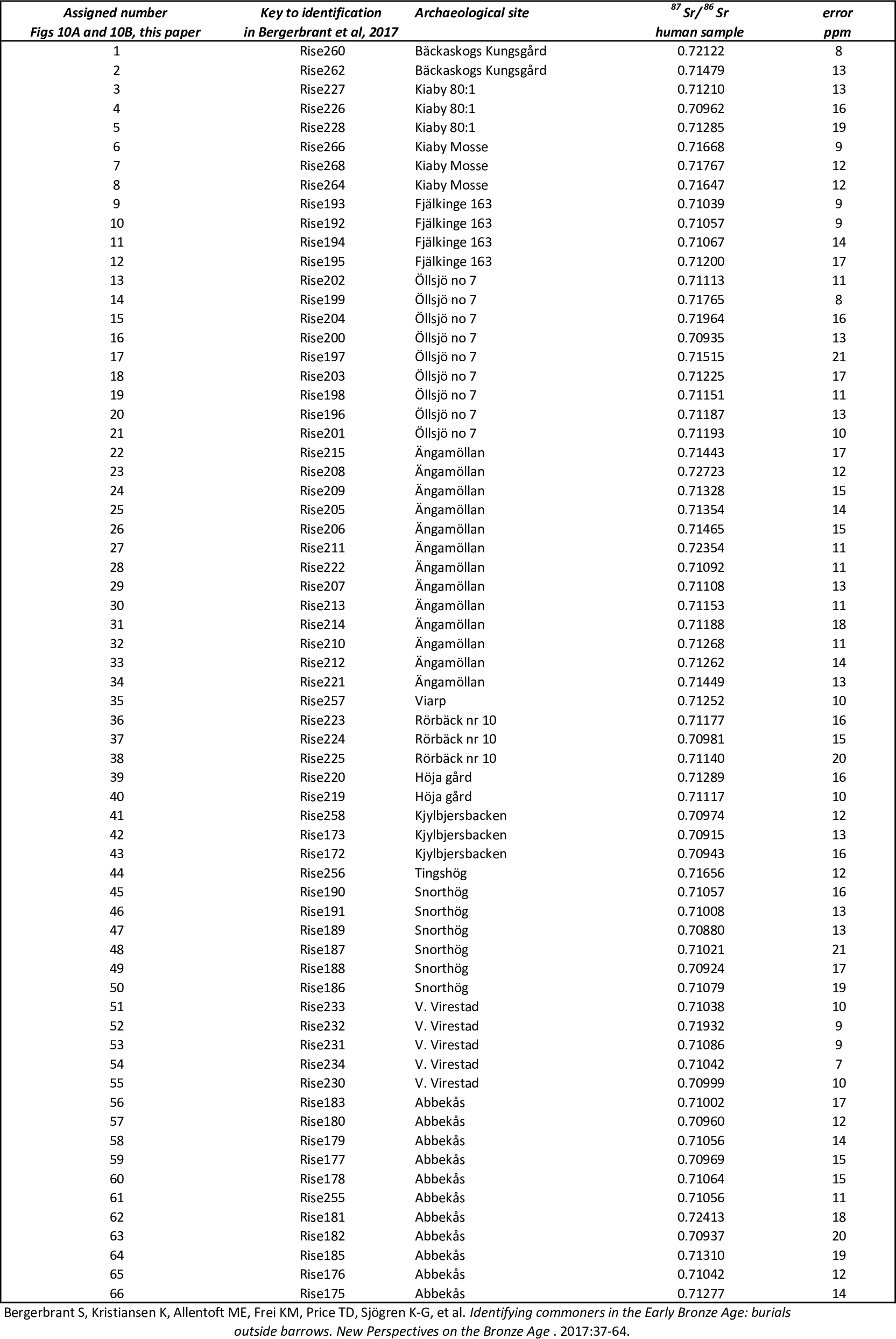

Supplement: S1 Table — (TIF) [file pone.0250279.s004.tif]
