# Supplementary material for: Testing Late Bronze Age mobility in southern Sweden in the light of a new multi-proxy strontium isotope baseline of Scania
Source: PLoS One. 2021 Apr 21;16(4):e0250279. doi: 10.1371/journal.pone.0250279 (PMC8059841; doi:10.1371/journal.pone.0250279)
Supplement: S1 File — (DOCX) [file pone.0250279.s005.docx]

*Supporting information*

*for*

Testing Late Bronze Age mobility in southern Sweden in the light of a new multi-proxy strontium isotope baseline of Scania (Sweden)

Ladegaard-Pedersen, Pernille^1^, Sabatini, Serena^2^, Frei, Robert^3^, Kristiansen, Kristian^2,4^ and Frei, Karin Margarita^1^.

^1^National Museum of Denmark, Copenhagen, Denmark.

^2^ Department of Historical Studies, University of Gothenburg, Gothenburg, Sweden

^3^ Department of Geosciences and Natural Resource Management, University of Copenhagen, Copenhagen, Denmark

^4^Globe Institute, Lundbeck Foundation, GeoGenetics Centre, Copenhagen, Denmark

## Site-specific variations of the different proxies across Scania

We investigated site-specific variations of the different proxies across Scania and within the identified Areas 1-4. In doing so, we computed site-specific absolute differences (delta; Δ^87^Sr/^86^Sr) in strontium isotopic ratios of the three proxies from individual measurements from sites with multi-proxy sampling (Table 1). These are shown diagrammatically (S3 Fig). Excluding the very high and removed plant sample of site 4 (as refereed earlier), the average, absolute Δ^87^Sr/^86^Sr between plant and soil leachates is Δ^87^Sr/^86^Sr =0.0016±0.0019 (1σ), with a maximum difference (Δ^87^Sr/^86^Sr=0.0081) found at site 17 within Precambrian granite, and a minimum difference (Δ^87^Sr/^86^Sr =0.00004) at site 13 within Triassic mud-clay-siltstone. The average Δ^87^Sr/^86^Sr between plant and stream water samples is Δ^87^Sr/^86^Sr=0.0017±0.0020 (1σ), with a maximum difference (Δ^87^Sr/^86^Sr =0.0070) found at site 12 within Silurian shale, and a minimum difference (Δ^87^Sr/^86^Sr =0.0001) at site 1 within Tertiary limestone. The average difference between stream water samples and soil leachates is Δ^87^Sr/^86^Sr=0.0019±0.0015 (1σ), with a maximum difference (Δ^87^Sr/^86^Sr =0.0053) found at site 18 within Precambrian granodioritic-granitic gneiss, and a minimum difference (Δ^87^Sr/^86^Sr=0.0001) at site 21 within Triassic arkose (sandstone). There is no obvious pattern of the Δ^87^Sr/^86^Sr values and affinity to e.g. bedrock type, nor is there a discernible pattern of the Δ^87^Sr/^86^Sr values between the different proxy types. This is in contrast to findings in Cyprus, where contrasting patterns of site-specific Δ^87^Sr/^86^Sr values where found in sedimentary vs crystalline areas (Ophiolites), the former showing consistent patterns of the site-specific Δ^87^Sr/^86^Sr values between proxies, with Δ-values up to 0.0008, the later showing larger differences and irregular patterns, and Δ-values up to 0.0019 (1), which, however, still is much more homogenous across proxy archives, than in the present study within Scania. With the exception of the Δ^87^Sr/^86^Sr value between stream water and plant sample of site 12, there seems to be a tendency for the absolute Δ^87^Sr/^86^Sr values from Areas 2-4 to be ≤0.0041, while the Δ^87^Sr/^86^Sr values from Area 1 are higher, up to 0.0081, and more scattered (S1 Fig), indicating the geological complexity of the Precambian Terranes to reflect in the intra-site proxy archive Δ^87^Sr/^86^Sr values. These Δ^87^Sr/^86^Sr values compares to the intra-site proxy differences between soil leachates and plant samples reported in the IRHUM study in France, with the largest absolute Δ-values up to 0.00847, and with average Δ-values of 0.0008 ± 0.0012 (2). Intra-site proxy differences have been reported by several other studies (3-6), including a recent study from Ireland sampling different plant types at each site, e.g. grasses, bushes and trees, found intra-site variations to vary from Δ^87^Sr/^86^Sr <0.0001 to 0.0052, indicating the intra-site variation to be not related solely to proxy type, but also illustrating a general intra-site variation to be expected within biosphere proxy archives (7).


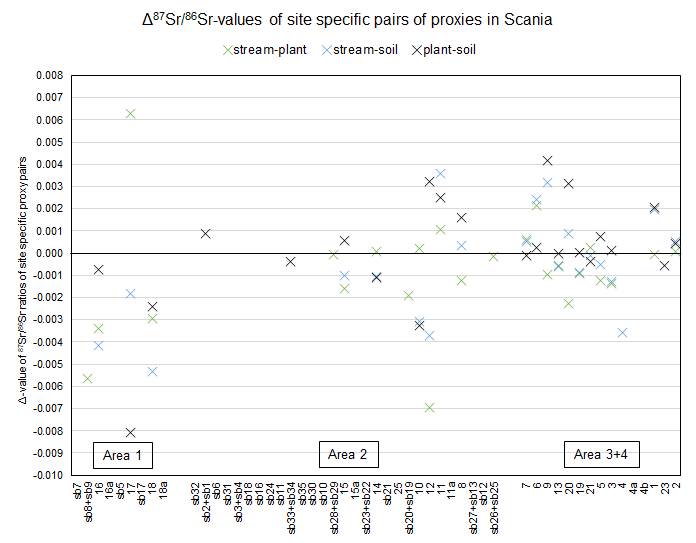


**S1 Fig Delta values (Δ^87^Sr/^86^Sr) of strontium isotopic ratios of site-specific pairs of proxies.**

## Comparison of baselines with a previously defined, faunal baseline for Scania

Modern and archaeological fauna data from sites in Scania, compiled and reported by Arcini (8), show a range of strontium isotopic compositions from ^87^Sr/^86^Sr = 0.709 - 0.719, and define a Scania-wide faunal baseline of ^87^Sr/^86^Sr = 0.712 ± 0.004 (n=46, 2σ). It is constructed on the basis of data from enamel of small rodents, cattle, pigs and dogs. Fauna data from small, local rodents were previously proposed as suitable archives for the definition of baselines, with the idea in mind that these animals provide an averaged out biosphere strontium isotope signature (with plants and water as the main Sr sources in their diets) over small territories they thrive in (4, 9-11). This data is compatible, albeit with a smaller range, with the outlier-screened Scania wide baseline proposed herein defined as ^87^Sr/^86^Sr = 0.7133 ± 0.0059 (n=102, 2σ). The fauna-based data of Arcini (8) are too few to group into the sub-areas proposed herein, and therefore we are unable to diversify the and directly compare this data set to our sub-area baselines defined above.

Strontium isotope distributions in Scania-wide and sub-regional multi-proxy bioavailable Sr baselines

The use of strontium isotopes in provenance and mobility studies in archaeology, food authenticity studies and in other similar studies, relies on the comparison between the strontium isotope ratios of the material under investigation, and the strontium isotope ratios defining the potential area of origin. Defining the strontium isotope range of a region is not a trivial task, and various approaches are applied. Applications range from using only the strontium isotopic ratios of archaeological human and faunal remains from a specific archaeological sites under investigation to using modern environmental samples (soil, water and plants) from larger areas/regions surrounding an archaeological site of interest, just to mention a few. Knowledge of the geology of a specific target area has shown to be essential in many cases (11-18). Characterizing the strontium isotope ranges of larger, perhaps geologically complex, areas, requires high density sampling which is not always achievable, and this has led to studies using geo-statistical modelling (17-21), geospatial modelling (14, 22) or domain mapping (1, 11, 13, 22) of sample populations to construct spatial distribution maps Sr isotope ratios that are based on environmental proxy archive samples across regions or countries of interest. Such maps and baselines can be used in combination with specific data from site-near samples to interpret prehistoric mobility (17, 23-26). Geographical criteria, such as distance from archaeological sites, have also been used to define which samples to include when defining the range of bioavailable strontium isotope signatures that are relevant for specific investigations (27, 28).

In the present study, taking into account the large regional differences of biosphere Sr isotope data, we found it appropriate to propose to construct sub-regional, area-specific baselines, as defined in the previous section, for the use as discriminators for local *vs* non-local provenance of materials of interest, including human archaeological finds. The difference between a Scania-wide and a sub-regional approach is illustrated on Figs S2 and S3, where Scania-wide baselines and sub-regional baselines (Areas 1, 2 and 3-4) are depicted alongside the single data points used to constrain the baselines, which are defined by the mean ± 2σ of the relevant ^87^Sr/^86^Sr data populations (Tables 3 and 4). Fig S2 shows Scania-wide, multi-proxy and single-proxy baselines calculated from all the environmental ^87^Sr/^86^Sr values across Scania. The Scania-wide surface water baseline is statistically the narrowest baseline of all proxy archive based baselines, possibly reflecting an averaging out of strontium isotope compositions from various sources in the watersheds and respective catchment areas. The Scania-wide plant baseline defines the highest mean, but has a lower range than the widest single-proxy baseline, which is defined by soil leachates. The lower limit of the soil leachate single proxy baseline is at ^87^Sr/^86^Sr = 0.7051, a value which is much lower than the minimum measured strontium isotope ratio in this study. This indicates that the soil leachate baseline either is calculated from too few samples, or that the soil leachate data population is not normally distributed. The wide spread in soil leachate isotope signatures is an expression of the vastly different geological terranes in Scania, and we deem it inaccurate and unrealistic to define a single baseline valid for entire Scania based on soil leachate data only. From Fig S2 it is evident, that the large range and variation of the measured bioavailable strontium isotopic ratios across the terranes of Scania cannot be defined within a Scania-wide baseline, without leaving most areas misrepresented be the constraints of a Scania-wide baseline.

While statistically still overlapping to certain degree, baselines from Area 1, 2 and from combined Area 3-4 show marked peculiarities (Fig S3). The expanded baseline of Area 1, where the calculated single-proxy baselines defined as mean ^87^Sr/^86^Sr ± 2σ exceeds the range of measured proxies, likely indicates the sampling density being too low to fully encompass the strontium isotope variations found within these terranes. This is especially the case with the plant baseline, which greatly exceeds the range of the single data points. The spread in ^87^Sr/^86^Sr signatures indicates a complex mixing of Sr from different sources, particularly of the significantly different bedrocks with a wide range of geological ages in this Precambrian area. Area 1 is characterized by an enhanced heterogeneity of proxy archive data, also intra-site-wise. Data from site 17 are an extreme case where there is no agreement between water, soil leachate and plant data (Fig S3). While divergence of plant and soil data from water data can be explained by the stream water carrying a transported and averaged out signatures from a heterogeneous catchment area with respect to Sr sources, we are unable at this stage to explain why soil leachate and plant signatures from site 17 lie so far apart from each other. The baseline characterization of Area 1 in particular awaits additional multi-proxy archive analyses, and an in-detail study which addresses the causes for the large intra-site variations of our data. While Area 1 is dominated by crystalline, Precambrian rock, the defined multi-proxy baseline (mean ^87^Sr/^86^Sr= 0.7184 ± 0.0061 (2σ; n=16); Table 4) is compliant, however still somewhat lower, with the baseline defined from the areas of crystalline, Precambrian terrane surrounding the area of Falbygden (Fig 1), with a baseline defined by a mean ^87^Sr/^86^Sr= 0.7220 ±0.0032 (1σ; n=39)(22). The baseline of ^87^Sr/^86^Sr = 0.7335 ± 0.0070 (1σ; n=4) reported from the more northerly site within Mälardalen (29) (Fig 1), is higher than what we define for the Area. These discrepancies add further support to our suggestion to apply the multi-proxy baseline of Area 1 (and similar terranes) with caution, just as even more caution is needed if relying on single-proxy data. Further more detailed sampling of different proxies is necessary to better constrain the baseline of this northern area in Scania, and additional baseline samples from archaeological sites should preferably be included in future studies on mobility within these areas. Looking into other studies from Europe, areas with similar, albeit not entirely identical, strontium isotope ranges as within Area 1 are reported from areas within the massifs of France, as well as areas within the Vosges, the Pyrenees, western part of the Iberian peninsula, parts of the Italian and Austrian Alps, as well as smaller parts of northern England, Ireland and eastern Europe (7, 13, 17, 18, 20).

The multi-proxy baseline of Area 2 defined as mean ^87^Sr/^86^Sr = 0.7140 ± 0.0043 (2σ, n=48) (Table 4), is constrained by more sampling sites, where the single-proxy baselines correspond better with each other, compared with those in Area 1 (Fig 8B). We note that all single-proxy baselines of Area 2 are in themselves consistent and compatible with each other, yet with varying scatter, while this is not the case for Area 1. The calculated, single-proxy water baseline of Area 2 is the narrowest baseline, with a range identical to the ranges defined by the measured data. However, the calculated single-proxy plant baseline, and the calculated single-proxy soil leachate baseline show constraints exceeding the majority of the measured data points within each proxy archives. From this, it is advisable to use multi-proxy data to constrain the baseline of Area 2 as a conservative choice, and additional baseline samples from archaeological sites should preferably be included in future studies on mobility within these areas. From the geologically similar area of Falbygden, north of Scania (Fig 1), the reported baselines from the areas dominated by Paleozoic sedimentary bedrocks of mean ^87^Sr/^86^Sr = 0.7146 ±0.0014 (1σ; n=45; water and fauna) (22) is similar to the multi-proxy baseline defined from Area 2. Also, the reported baseline of from the island of Öland (Fig 1), dominated by Paleozoic limestone, and with a mean ^87^Sr/^86^Sr = 0.7140 ±0.0024 (1σ; n=25; fauna) (29), is compliant with Area 2 of Scania, despite the more diverse geology of Area 2 (Fig 6), while the reported baseline from the island of Gotland, also dominated by Paleozoic bedrocks, is somewhat lower (mean ^87^Sr/^86^Sr = 0.7120 ± 0.0018 (1σ; n=26; soil and fauna) (29, 30)) and more similar to Area 3-4 of Scania, which is dominated by both Paleozoic, Mesozoic and Cenozoic sedimentary bedrock. This supports our approach for a construction of baselines that rely on multi-proxy data to best and adequately cover the biosphere range of the regions.

Baseline characteristics of Area 3-4 show a lower overall variation and scatter of the proxy data, with single-proxy surface water- and plant-baselines having constraints almost similar to the multi-proxy baseline with a mean of ^87^Sr/^86^Sr = 0.7110 ± 0.0030 (2σ, n=39) (Table 4), and covering similar ranges as the measured data (Fig 9B), albeit the surface water data still constrains the narrowest baseline. Only the soil leachate baseline shows a calculated lower limit largely exceeding the measured data, indicating that a single-proxy baseline relying on only soil leachates, is not adequate enough to constrain a reliable baseline with the sampling density of the present study. The baseline of Area 3-4 is defined by the most unradiogenic, i.e. lowest baseline limit of all areas, and this is likely to reflect the increased content of limestone-derived Sr from either the bedrock themselves, and/or from increased clastic carbonate components in the overlying tills in this area. The comparatively good agreement of individual proxy baselines calculated for Areas 3-4 compared to Areas 1 and 2 possibly lies in the nature of the bedrock in this area. The predominantly calcareous sediments underlying the clayey, carbonate rich Baltic tills are a strong buffering system for Sr and its isotope composition against the radiogenic biosphere components that derive from relatively Sr-poor bedrock and sandy tills that prevail in the other areas. This is a pattern also seen in a recent study in Cyprus (1), where single-proxy baseline differences were minimal in carbonate bedrock terranes, and more pronounced in crystalline, ophiolite bedrock-dominated terranes in the country. The multi-proxy baseline for Area 3-4 which is very much compatible with the baseline established for neighbouring country Denmark (excluding Bornholm), currently defined as ^87^Sr/^86^Sr =0.7096 ± 0.0016 (2σ) (14). Also, the baseline established for the Netherlands using archaeological fauna (31) is compatible with the strontium isotopic range of Area 3-4, and these ranges are commonly found within the sedimentary basins of Europe, both in site-specific studies in e.g. Germany, Hungary and Italy to mention a few (4, 27, 32) and in broader studies, e.g. (13, 17, 18, 20).


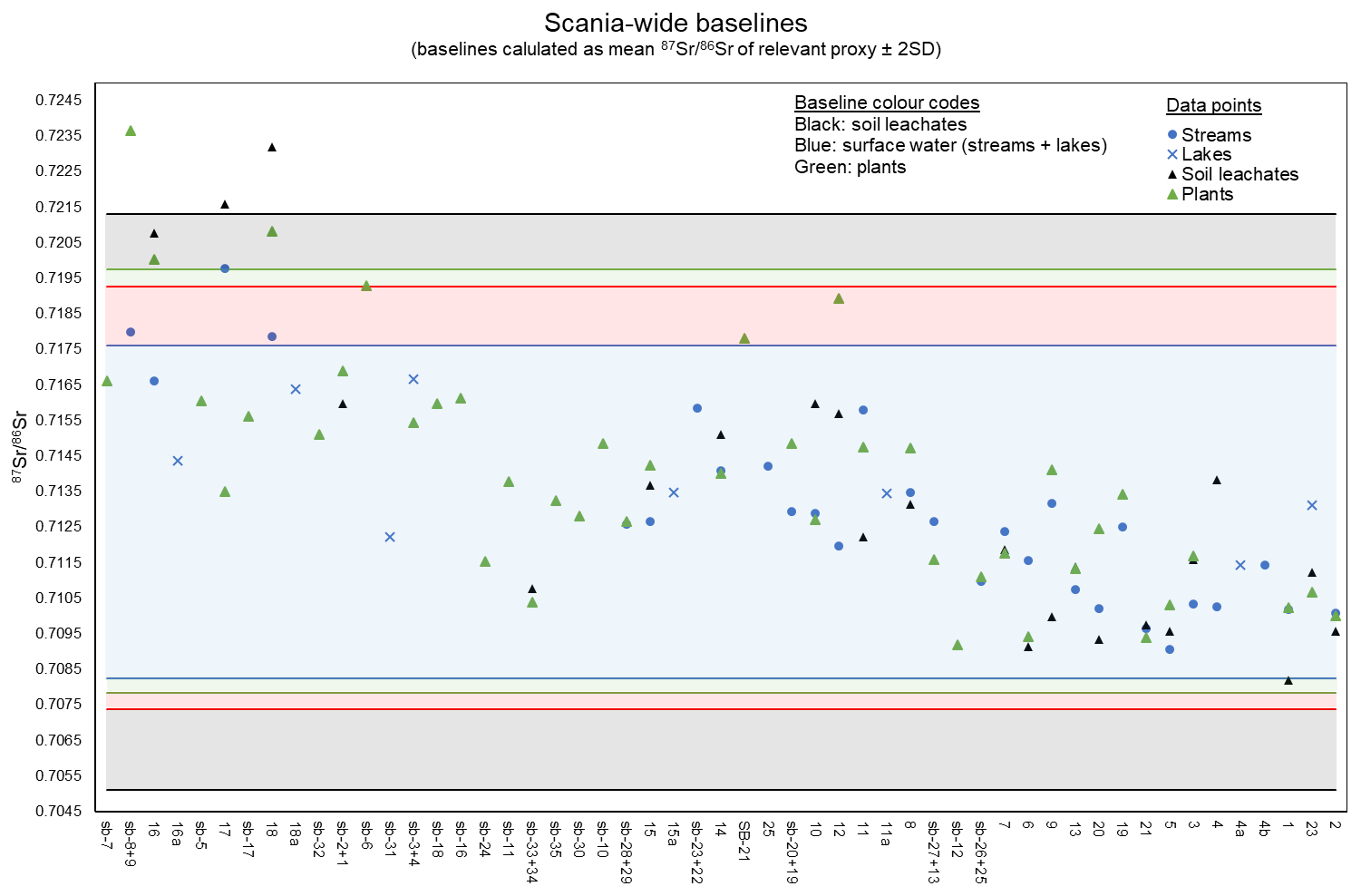


**S2 Fig Scania- wide baselines calculated from environmental proxies.** Sampling sites are shown on x-axis. Environmental strontium isotope ratios included in baseline calculations are shown. All baselines calculated as the mean ^87^Sr/^86^Sr ± 2σ of the relevant samples.


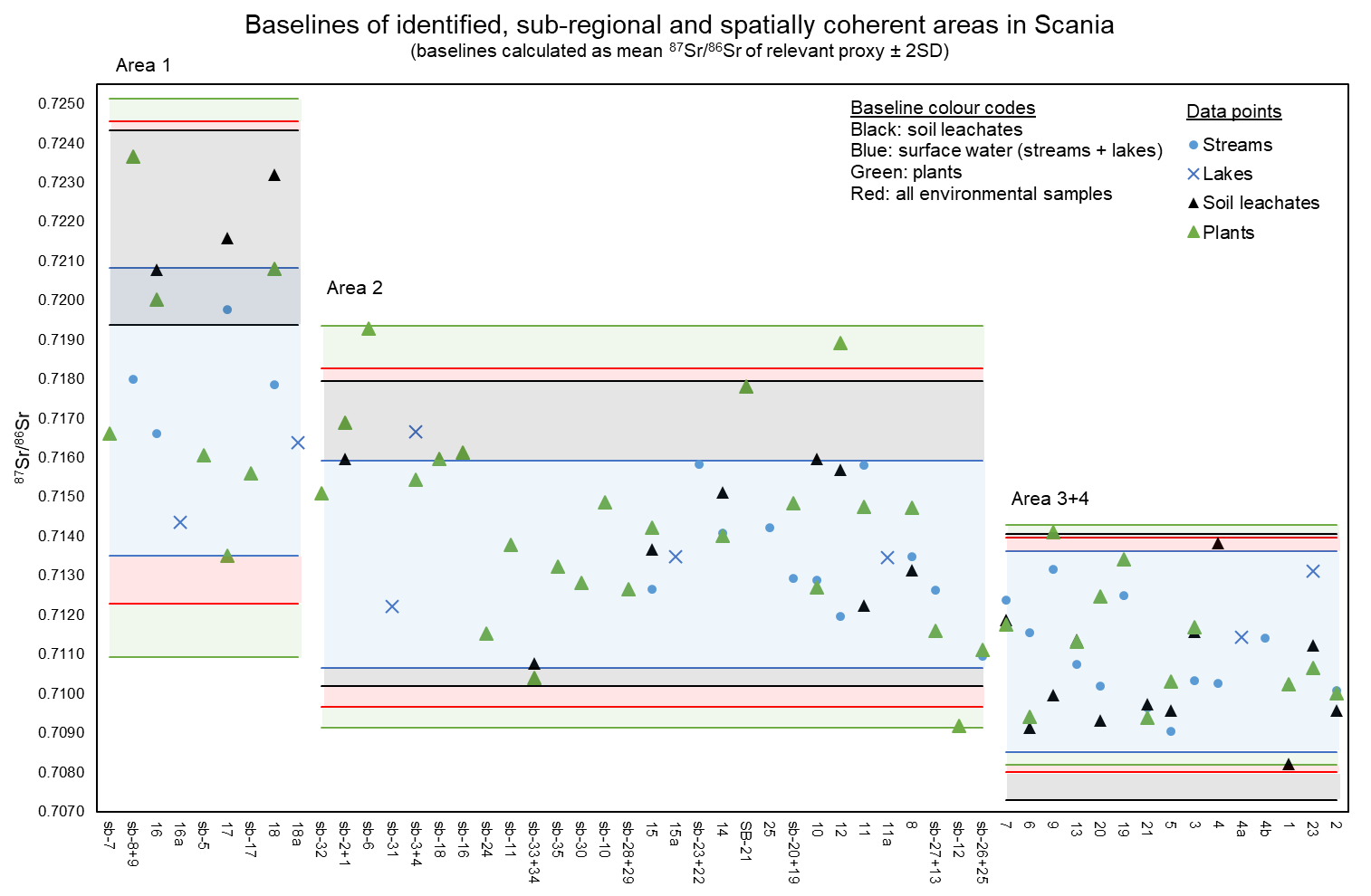


**S3 Fig Sub-regional baselines calculated from environmental proxies.** Diagram depicting both multi-proxy and single-proxy baselines of Area 1, Area 2, and Area 3-4. Sampling sites shown on x-axis. Environmental strontium isotope ratios included in baseline calculations are shown. All baselines calculated as the mean ^87^Sr/^86^Sr ± 2σ of the relevant samples.

**S1 Table Key to the identification of human samples from previous studies showed in Figs 10A and 10B.**

**

**

## References

1. Ladegaard-Pedersen P, Achilleos M, Dörflinger G, Frei R, Kristiansen K, Frei KM. A strontium isotope baseline of Cyprus. Assessing the use of soil leachates, plants, groundwater and surface water as proxies for the local range of bioavailable strontium isotope composition. Science of The Total Environment. 2020;708:134714.

2. Willmes M, McMorrow L, Kinsley L, Armstrong R, Aubert M, Eggins S, et al. The IRHUM (Isotopic Reconstruction of Human Migration) database &ndash; bioavailable strontium isotope ratios for geochemical fingerprinting in France. Earth Syst Sci Data. 2014;6(1):117-22.

3. Blum JD, Taliaferro EH, Weisse MT, Holmes RT. Changes in Sr/Ca, Ba/Ca and 87Sr/86Sr ratios between trophic levels in two forest ecosystems in the northeastern U.S.A. Biogeochemistry. 2000;49(1):87-101.

4. Maurer A-F, Galer SJG, Knipper C, Beierlein L, Nunn EV, Peters D, et al. Bioavailable 87Sr/86Sr in different environmental samples — Effects of anthropogenic contamination and implications for isoscapes in past migration studies. Science of The Total Environment. 2012;433:216-29.

5. Reynolds AC, Quade J, Betancourt JL. Strontium isotopes and nutrient sourcing in a semi-arid woodland. Geoderma. 2012;189-190(Supplement C):574-84.

6. Brönnimann D, Knipper C, Pichler SL, Röder B, Rissanen H, Stopp B, et al. The lay of land: Strontium isotope variability in the dietary catchment of the Late Iron Age proto-urban settlement of Basel-Gasfabrik, Switzerland. Journal of Archaeological Science: Reports. 2018;17:279-92.

7. Snoeck C, Ryan S, Pouncett J, Pellegrini M, Claeys P, Wainwright AN, et al. Towards a biologically available strontium isotope baseline for Ireland. Science of The Total Environment. 2020;712:136248.

8. Arcini C. The Viking Age: A Time with Many Faces: Oxbow Books; 2018.

9. Price TD, Burton JH, Bentley RA. The Characterization of Biologically Available Strontium Isotope Ratios for the Study of Prehistoric Migration. Archaeometry. 2002;44(1):117-35.

10. Grimstead DN, Nugent S, Whipple J. Why a Standardization of Strontium Isotope Baseline Environmental Data Is Needed and Recommendations for Methodology. Advances in Archaeological Practice. 2017;5(2):184-95.

11. Evans JA, Montgomery J, Wildman G. Isotope domain mapping of <sup>87</sup>Sr/<sup>86</sup>Sr biosphere variation on the Isle of Skye, Scotland. Journal of the Geological Society. 2009;166(4):617-31.

12. Hodell DA, Quinn RL, Brenner M, Kamenov G. Spatial variation of strontium isotopes (87Sr/86Sr) in the Maya region: a tool for tracking ancient human migration. Journal of Archaeological Science. 2004;31(5):585-601.

13. Evans JA, Montgomery J, Wildman G, Boulton N. Spatial variations in biosphere <sup>87</sup>Sr/<sup>86</sup>Sr in Britain. Journal of the Geological Society. 2010;167(1):1-4.

14. Frei KM, Frei R. The geographic distribution of strontium isotopes in Danish surface waters – A base for provenance studies in archaeology, hydrology and agriculture. Applied Geochemistry. 2011;26(3):326-40.

15. Ladegaard-Pedersen P, Achilleos M, Dörflinger G, Frei R, Kristiansen K, Frei KM. A strontium isotope baseline of Cyprus. Assessing the use of soil leachates, plants, groundwater and surface water as proxies for the local range of bioavailable strontium isotope composition. Science of The Total Environment. 2019:134714.

16. Hartman G, Richards M. Mapping and defining sources of variability in bioavailable strontium isotope ratios in the Eastern Mediterranean. Geochimica et Cosmochimica Acta. 2014;126(Supplement C):250-64.

17. Willmes M, Bataille CP, James HF, Moffat I, McMorrow L, Kinsley L, et al. Mapping of bioavailable strontium isotope ratios in France for archaeological provenance studies. Applied Geochemistry. 2018;90:75-86.

18. Hoogewerff JA, Reimann C, Ueckermann H, Frei R, Frei KM, van Aswegen T, et al. Bioavailable 87Sr/86Sr in European soils: A baseline for provenancing studies. Science of The Total Environment. 2019.

19. Bataille CP, von Holstein ICC, Laffoon JE, Willmes M, Liu X-M, Davies GR. A bioavailable strontium isoscape for Western Europe: A machine learning approach. PLOS ONE. 2018;13(5):e0197386.

20. Voerkelius S, Lorenz GD, Rummel S, Quétel CR, Heiss G, Baxter M, et al. Strontium isotopic signatures of natural mineral waters, the reference to a simple geological map and its potential for authentication of food. Food chemistry. 2010;118(4):933-40.

21. Bataille CP, Bowen GJ. Mapping 87Sr/86Sr variations in bedrock and water for large scale provenance studies. Chemical Geology. 2012;304:39-52.

22. Blank M, Sjögren K-G, Knipper C, Frei KM, Storå J. Isotope values of the bioavailable strontium in inland southwestern Sweden—A baseline for mobility studies. PLOS ONE. 2018;13(10):e0204649.

23. Madgwick R, Grimes V, Lamb AL, Nederbragt AJ, Evans JA, McCormick F. Feasting and Mobility in Iron Age Ireland: Multi-isotope analysis reveals the vast catchment of Navan Fort, Ulster. Scientific Reports. 2019;9(1):1-14.

24. Price TD, Frei Karin M, Dobat Andres S, Lynnerup N, Bennike P. Who was in Harold Bluetooth's army? Strontium isotope investigation of the cemetery at the Viking Age fortress at Trelleborg, Denmark. Antiquity. 2015;85(328):476-89.

25. Frei KM, Villa C, Jørkov ML, Allentoft ME, Kaul F, Ethelberg P, et al. A matter of months: High precision migration chronology of a Bronze Age female. PLOS ONE. 2017;12(6):e0178834.

26. Frei KM, Mannering U, Kristiansen K, Allentoft ME, Wilson AS, Skals I, et al. Tracing the dynamic life story of a Bronze Age Female. Scientific reports. 2015;5:10431.

27. Cavazzuti C, Skeates R, Millard AR, Nowell G, Peterkin J, Bernabò Brea M, et al. Flows of people in villages and large centres in Bronze Age Italy through strontium and oxygen isotopes. PLOS ONE. 2019;14(1):e0209693.

28. Snoeck C, Pouncett J, Ramsey G, Meighan IG, Mattielli N, Goderis S, et al. Mobility during the Neolithic and Bronze Age in Northern Ireland explored using strontium isotope analysis of cremated human bone. American journal of physical anthropology. 2016;160(3):397-413.

29. Wilhelmson H, Ahlström T. Iron Age migration on the island of Öland: Apportionment of strontium by means of Bayesian mixing analysis. Journal of Archaeological Science. 2015;64:30-45.

30. Fraser M, Sanchez-Quinto F, Evans J, Storå J, Götherström A, Wallin P, et al. New insights on cultural dualism and population structure in the Middle Neolithic Funnel Beaker culture on the island of Gotland. Journal of Archaeological Science: Reports. 2018;17:325-34.

31. Kootker LM, van Lanen RJ, Kars H, Davies GR. Strontium isoscapes in The Netherlands. Spatial variations in 87Sr/86Sr as a proxy for palaeomobility. Journal of Archaeological Science: Reports. 2016;6:1-13.

32. Alt KW, Knipper C, Peters D, Müller W, Maurer A-F, Kollig I, et al. Lombards on the move–an integrative study of the migration period cemetery at Szólád, Hungary. PloS one. 2014;9(11).
